# High gas barrier coating using non-toxic nanosheet dispersions for flexible food packaging film

**DOI:** 10.1038/s41467-019-10362-2

**Published:** 2019-06-11

**Authors:** Jingfang Yu, Kanittika Ruengkajorn, Dana-Georgiana Crivoi, Chunping Chen, Jean-Charles Buffet, Dermot O’Hare

**Affiliations:** Chemistry Research Laboratory, Department of Chemistry, University of Oxford, 12 Mansfield Road, Oxford, OX1 3TA UK

**Keywords:** Synthesis and processing, Two-dimensional materials

## Abstract

One of the major challenges in the circular economy relating to food packaging is the elimination of metallised film which is currently the industry standard approach to achieve the necessary gas barrier performance. Here, we report the synthesis of high aspect ratio 2D non-toxic layered double hydroxide (LDH) nanosheet dispersions using a non-toxic exfoliation method in aqueous amino acid solution. High O_2_ and water vapour barrier coating films can be prepared using food safe liquid dispersions through a bar coating process. The oxygen transmission rate (OTR) of 12 μm PET coated film can be reduced from 133.5 cc·m^−2^·day^−1^ to below the instrument detection limit (<0.005 cc·m^−2^·day^−1^). The water vapour transmission rate (WVTR) of the PET film can be reduced from 8.99 g·m^−2^·day^−1^ to 0.04 g·m^−2^·day^−1^ after coating. Most importantly, these coated films are also transparent and mechanically robust, making them suitable for flexible food packing while also offering new recycling opportunities.

## Abstract (web summary)

Food packaging industry is in search of non-toxic, stable, and recyclable coatings acting as gas-barriers. Here the authors synthesise transparent films meeting these requirements, based on high aspect ratio layered double hydroxide nanosheets obtained by exfoliation in aqueous amino acid solution.

## Introduction

There is an urgent unmet need for scalable solutions that can replace metallised film with a solution-deposited or coextruded layer that can be recycled without separation from the rest of the packaging materials. Two-dimensional (2D) inorganic nanosheets are impermeable to gas molecules so exploiting their potential for high aspect ratio platelet structures is an effective approach to construct tortuous pathways to lower molecular diffusion rates through polymeric materials. The key challenges for high gas barrier food packaging materials are as follows (i) the preparation of stable, high aspect ratio non-toxic 2D nanosheet dispersions and (ii) the use of an existing commercial coating technique.

Barrier coatings containing dispersions of two-dimensional (2D) inorganic materials can be used to prepare protective layers against oxygen, moisture, chemicals, electricity, and harsh environments. A large variety of methods have been reported to enable the exfoliation of layered materials, the current state-of-the-art approach involves exfoliation in solvents^1^ or intercalation-exfoliation process^2^.

However, the production of an oxygen barrier coated film that is comparable to metalised polymer film is urgently required to address the many challenges of the circular economy for plastic use. As this film will be in contact with food, this imposes severe restrictions on the type of chemistry that can be deployed,. Furthermore, it also must be a drop-in solution for existing coating technologies and be cost effective compared to aluminium vapourisation. As a result, the production of scalable, environmentally friendly, non-toxic, high aspect ratio two-dimensional nanosheet coating to produce oxygen barrier food packaging film still remains an unsolved major challenge.

The exfoliation of naturally occurring platelet clay-like materials such as montmorillonite in water has already been extensively investigated as a possible film coating technology^3–5^ for the food packaging industry. However, for these naturally-sourced materials there still remains major concerns regarding migration of uncontrolled compositional impurities (such as heavy metals).

Layered Double Hydroxides (LDHs) are a large class of lamellar anionic synthetic clays that are commonly represented by the formula, [M_1–*x*_M’_*x*_(OH)_2_]^*a*+^[A^*n*−^_*a*/*n*_]•*m*H_2_O (M and M’ are commonly divalent and trivalent cations respectively, A^*n*−^ is the interlayer anion, and 0 < *x* < 1). The first LDH was termed hydrotalcite, it was found in a Norwegian geological specimen, and was subsequently chemically identified as [Mg_0.75_Al_0.25_(OH)_2_][CO_3_]_0.125_ (Mg_3_Al-CO_3_-LDH). Today MgAl-LDHs can be fully synthetic good manufacturing practice (GMP) materials, some sterile pharmaceutical grades have regulatory approvals both as medicines and food contact materials^6^.

However, the synthesis of LDH nanosheets is extremely demanding, the very high in-plane charge-density that means strong interlayer electrostatic interactions need to be overcome. Top down approaches have been successful, typically requiring exfoliation in very high dielectric constant liquids such as formamide driven by mechanic shearing/sonication^7^. Bottom-up approaches in which LDH nanosheets are grown within the water pool of an inverse oil-in-water emulsion have also been successful but this technology is very low yielding generating large amounts of solvent and surfactant waste streams which effectively limits its scalability^8^.

Amino acids have been employed to exfoliate LDHs in formamide^9^, the amino acids were considered as an intercalant that attracts a large amount of formamide to penetrate the LDH layers breaking the strong interlayer electrostatic interactions leading to exfoliation. Previous report^10^ indicates that ZnAl-LDH can be exfoliated in 1.5 M l-serine solution by agitation and ultrasonication treatment. However, as far as we are aware, amino acids have not been previously used in exfoliation of LDH by calcination and reconstruction method.

Our approach uses a totally synthetic platelet layered hydroxide materials platform that allows us to comply with GMP certification and that is already generally recognised as safe (GRAS) and approved for human^6^. As a result, we consider MgAl-LDHs to be an ideal layered materials platform to form the basis of a new barrier technology for food packaging applications. Here we report the use of aqueous amino acids solutions to prepare stable, high aspect ratio LDH nanosheet dispersions. Our approach combines the use of the well-known memory effect^11^ to reconstruct LDHs in water and the high dielectric increment^12^ of zwitterionic amino acids to generate very high dielectric aqueous solutions (Supplementary Table 1). We report the synthesis of high aspect ratio 2D non-toxic LDH nanosheets that can be stably dispersed in water. Using these high aspect ratio LDH nanosheets as a coating layer on PET substrate, we are able to decrease OTR of PET from 133.5 cc m^−2^ per day to below 0.005 cc m^–2^ per day. Similarly, WVTR of the PET film can be decreased from 8.99 to 0.04 g m^–2^ per day.

## Results

### Synthesis of LDH nanosheets and its mechanism

**Fig. 1:**
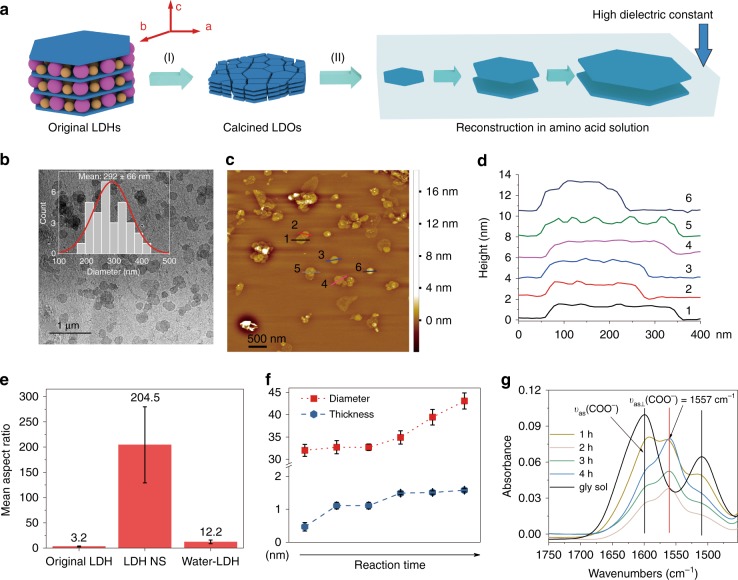
High aspect ratio LDH nanosheets. **a** Schematic showing (I) calcination (interlayer water and anions are removed by calcination) and (II) reconstruction process and the preferential growth inhibition in a high dielectric constant solution: thickness growth is much slower than the diameter growth, giving high aspect ratio nanosheets. TEM image (**b**), AFM image (**c**) and height profiles (**d**) of the LDH NS reconstructed in glycine solution. The inset in TEM image represents the diameters measured by TEM. **e** Mean aspect ratio of the original LDH, LDH NS and control LDH reconstructed in water (Water-LDH). Aspect ratio was calculated by diameter divided by thickness of individual particles from AFM measurements of samples at more than three different spots. Error bar represents the standard deviation from more than 30 measurements. **f** Estimation of crystallite sizes calculated from Scherrer equation (Eq. (2)) confirming the significant growth inhibition along the *c*-axis. The red box with dotted line and the blue circle with dotted line represent the estimation of the diameter growth and thickness growth with reaction time. Error bar represents standard error in the curve fitting. **g** IR spectra: formation of hydrogen bonding evidenced by a red shift of the asymmetric vibration of COO^−^ group of glycine and that part of the group is shifted to orthogonal position (*ν*_*as*⊥_(COO^−^) = 1557 cm^−1^) during reconstruction in glycine solution. The black lines are assigned to the asymmetric vibration of COO^−^ group and the red line is attributed to the shift to orthogonal position of part of the group. Source data are provided as a Source Data file

A platelet morphology sample of Mg_2_Al-CO_3_-LDH was initially calcined in air at 450 °C for 12 h to give the layered double oxide (LDO). It is well known that an LDO prepared at this temperature is capable of regenerating back to the original crystalline Mg_2_Al-CO_3_-LDH by the addition of water in air. The CO_2_ initially lost upon calcination is recaptured from the atmosphere to create the charge balancing CO_3_^2−^ anions. This is a poorly understood phenomena termed the memory effect which was previously thought be partly topotactic in nature. However, we have discovered it is in fact a dissolution-recrystallisation process^13^ and so it can be used to control LDH platelet size and aspect ratio (Fig. 1a and METHOD). Dissolving amino acids in water is known to induce a large additional dielectric increment^12^ to the solution. So, when we add a 2 M aqueous glycine solution to Mg_2_Al-LDO and heat at 100 °C for 24 h, it produces a translucent gel (Supplementary Fig. 1). The gel can be diluted to produce an amino acid stabilised aqueous dispersion of high aspect ratio Mg_2_Al-LDH nanosheets (LDH NS) (Fig. 1b–d) (Supplementary Tables 2 and 3 and Supplementary Fig. 2). If we define aspect ratio as platelet diameter divided by platelet thickness, the LDH NS have a mean aspect ratio of 204.5 ± 75.4 (Fig. 1e), that is *ca*. 64 times higher than the original pre-calcined LDH sample (Supplementary Fig. 4a, b) and ca. 20 times higher than the aspect ratio of the control LDH reconstructed in pure water (Water-LDH) (Supplementary Fig. 4c, d). Using atomic force microscopy (AFM), we find the majority of the LDH NS platelets comprise of just two LDH layers (Fig. 1d and Supplementary Fig. 3a) (0.48 nm for each metal hydroxides layer) and glycine (0.3 nm), they also have a well define shape rather than random irregular fragments that are usually obtained by exfoliation of LDHs^7^.

We have investigated the mechanism of the reconstruction process in aqueous glycine. We find that the LDO dissolves rapidly at 100 °C in the concentrated acidic glycine solution (pH = 5.6 of 2 M glycine), which is then followed by an almost instantaneous reconstruction of the LDH structure (Supplementary Fig. 5). During the reconstruction process, while the LDO peak disappears after four minutes, crystalline LDH nanosheets nucleate after three minutes. The rate of LDH crystallisation along the platelet stacking axis is much slower, after 15 h of reaction very low crystallographic coherence along the platelet stacking axis (*c*-axis) can be observed as evidenced by the observation of a broad diffraction feature centred at around 2*θ* = 11.60° which is the position of the 003 Bragg reflection (Supplementary Fig. 6). It appears that in glycine solution LDH crystallisation in the *ab-*plane is promoted at the expense of the interlayer growth (*c*-axis). LDH crystallisation in *ab-*plane as well as along *c*-axis are inhibited due to its high dielectric constant: both the diameter and thickness of LDH NS are smaller compared to the control LDH reconstructed without glycine (Water-LDH) (Supplementary Fig. 3). However, the inhibition effect is much more significant along the *c*-axis **(**Fig. 1f; METHOD): giving high aspect ratio LDH NS. The presence of the zwitterionic amino acid produces an solution with a high dielectric constant (2 M glycine aqueous solution has a dielectric constant of 125) which is comparable to that of formamide (dielectric constant of 111) a well-known exfoliation agent for LDHs^12^. This medium both screens the electrostatic interactions decreasing the interactions between the positively charged LDH NS and counter anions (mainly CO_3_^2−^ under this condition) and also solvates and stabilises the positively charged nanosheets through hydrogen bonding (Fig. 1g and Supplementary Fig. 7). The reconstruction and stabilisation of LDH NS in concentrated aqueous glycine seems to be quite general, we have obtained a range of LDH NS containing various metal cations including NiAl, MgIn, MgGa, and ZnAl-LDH NS through the calcination and reconstruction method (Supplementary Fig. 8).

### Barrier coating film and characterisation

We can recover solid samples of the LDH nanosheets by precipitation from the aqueous suspension (Supplementary Fig. 9a) upon addition of NaOH solution followed by centrifugation. The precipitate can then be washed with water to remove the excess glycine and NaOH. Precipitation by this method leads to LDH platelet restacking as we observe increased intensity of the 003 Bragg reflection for the sample (Supplementary Fig. 6).

**Fig. 2:**
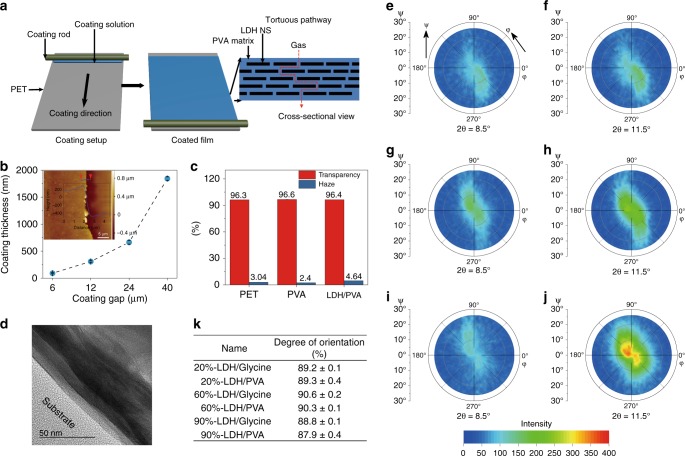
Structure of barrier films containing LDH nanosheets. **a** Schematic of coating process and tortuous pathway. The coating solution was spread and coat on the substrate PET film by a semi-automatic bar coater to form the aligned structure of LDH NS in PVA matrix. The well aligned impermeable LDH forces gas molecules to go through a tortuous pathway to diffuse through the film. **b** Coating layer thickness plotted as a function of coating gap. Error bar represents the standard deviations of more than 10 measurements. The inset shows the coating layer thickness of film coated with 24 μm coating gap measured by AFM. The coating solution used to coat all the films contains 3 wt% LDH NS and 2 wt% PVA. **c** Transparency and haze of the barrier films of PET, 10 wt% PVA coated PET, and 10 wt%-60% LDH coated PET films. **d** Cross-sectional TEM image of the barrier film containing 60% LDH showing ordered structure where LDH nansheets are aligned parallel to each other. Pole figure measurements of LDH/PVA phase and LDH/Glycine phase in the barrier films (all the films were coated with 24 μm gap) containing 20% (**e**, **f**), 60% (**g**, **h**) and 90% LDH (**i**, **j**) in the coating layer. **k** Summary of degree of orientation of LDH nanosheets calculated by Eq. (3). Error bar represents standard error from the curve fitting. The total solid content of all the coating solutions (except in **c**) were 5 wt% for all the coated film samples discussed in this figure. PVA with MW of 67,000 g mol^−1^ was used for all the coating films

In order to establish an effective gas barrier layer both theoretical studies^14^ and experimentation^15^ has shown that both aligned and high aspect ratio nanosheets are necessary in order to setup extra diffusion pathways (Fig. 2a) for the gas molecules to move around the impermeable NS. This tortuous pathway theory is a universal concept to gas barrier^16^, moisture barrier^3^, heat transfer^3^, molecular migration^17^ and ionic conductvity^18^ that is widely adopted in packaging materials, insulation materials and flame retardant materials.

To prepare a coating solution the LDH nanosheets gel was mixed with appropriate mount of a stock aqueous 10 wt% PVA (MW 67,000 g mol^–1^) solution, the dispersion was diluted with water to give the required total solid content (Supplementary Fig. 9b). 12 μm polyethylene terephthalate (PET) film was chosen as the film substrate, the LDH/PVA coating solution was applied in a single coating step (Fig. 2a) using a commercial bar coater. The dried coating layer thickness can be tuned by changing the coating rods with different coating gap (coating gap refers to the deposited wet film thickness, it is pre-determined by the wire diameter of the rods) (Fig. 2b and Supplementary Fig. 10 and 11). In our studies, the dried coating layer thickness was tuned from *ca*. 100 nm to 1.8 μm. We found that the coating layer did not decrease the transparency of the PET substrate nor the haze of the coated film (Fig. 2c). We analysed the thermal properties of our barrier films. The residues at 600 °C are 16.3, 17.3, and 23.6% for PET, PVA coated PET and PVA/LDH NS coated PET, respectively (Supplementary Fig. 12). All three films showed very similar temperature at the maximum decomposition rate (*T*_max_) of ca. 421 °C (Supplementary Table 4)^19^. The results suggest that our thin coating layer does not change the thermal degradation process of the bulk PET substrate film. Before *T*_max_, the PVA/LDH coated film showed reduced thermal stability probably due to the decomposition of glycine and loss of absorbed water. When the temperature is higher than *T*_max_, PVA/LDH coated film exhibited enhanced thermal stability due to the LDH layer heat insulation and barrier effect^19^.

We have evaluated the effectiveness of the coating layer by varying the total solid content (wt% LDH + wt% PVA) in the solution and the ratio of LDH to PVA. The coating solutions are labelled as X wt%-Y% LDH where X wt% (ranging from 3 to 13 wt%) refers to the total solid content (LDH and PVA) in water solution and Y% (ranging from 10 to 90%) refers to the weight ratio of LDH over PVA. When referring to the coated films, *Y*% is the weight ratio of LDH over PVA.

In the XRD patterns (Supplementary Fig. 13) of the dried coated PET films, we have identified Bragg reflections that we can assign to two LDH containing phases: an LDH containing variable amounts of intercalated PVA and a second LDH phase containing intercalated glycine (d-spacing is *ca*. 0.77 nm). The interlayer separation of the LDH/PVA intercalation phase decreases from 1.1 to 1.0 nm when the weight percentage of LDH nanosheets increases from 10 to 90% in the coating layer. We believe the decreasing interlayer separation is a result of less PVA present between individual layers in the LDH nanosheet when the percentage of LDH increases in the coating formulation.

The LDH nanosheets are well aligned parallel to the substrate PET film (Fig. 2d). The alignment of nanosheets within the film was examined by X-ray diffraction, pole figure measurements allow us to estimate the distribution of nanosheet orientations within the PVA matrix (Fig. 2e–j). Two sets of measurements were carried out at fixed 2*θ* degrees values of 8.5° (the LDH/PVA intercalated phase) and 11.5° (LDH/Glycine phase). Three samples (containing 20, 60, and 90% LDH) were scanned at a tilting angle (*ψ*) and a rotation angle (φ) (Fig. 2e). The pole figures show some anisotropic scattering but are all centred around 0° in *ψ* indicating that LDH layers are well aligned around (003) crystal plane (parallel to PET substrate, where scattering intensity at high *ψ* angle indicates the presence of LDH layers aligned *ψ* angle away from (003) crystal plane). We find the oriental distribution of the LDH/PVA sheets is very similar to that of the LDH glycine sheets. The oriental distribution is compared by the averaged full width at half maximum height (FWHM) of their scattering from all φ angles. To estimate the FWHM of the LDH NS orientation distribution the scattering from all φ angles was averaged. This allowed us to produce a single set of scattering intensities as a function of sample tilt ψ. These could then be fitted with a Gaussian distribution as shown below: where y_0_ is the background intensity, *A* is the area, *w* is the FWHM and *x*_c_ is the peak centre.

1 $$y = y_0 + \frac{{A{\mathrm{e}}^{\frac{{ - 4{\mathrm{ln}}(2)(x - x_{\mathrm{c}})^2}}{{w^2}}}}}{{w\sqrt {\frac{{\mathrm{\pi }}}{{4{\mathrm{ln}}(2)}}} }}.$$

The fitting gave FWHM (Supplementary Fig. 14a–c) of the LDH/Glycine and LDH/PVA phase of 19.5 ± 0.3° and 19.3 ± 0.6°, 17.0 ± 0.2° and 17.4 ± 0.2°, 20.1 ± 0.1° and 21.8 ± 0.8° for the coating films containing 20%, 60%, and 90% LDH nanosheets respectively (Supplementary Fig. 14d), suggesting that coating film containing 60% LDH has the highest degree of orientation of 90.3%, while the 90% LDH film has the lowest degree of orientation of 87.9% (Fig. 2k) (“Methods” section). The highest degree of preferred orientation is comparable to that observed in the literature^5,20^. The anisotropy of the FWHM for 60% LDH coating film was also determined. The pole figure data measured at 2*θ* = 8.5° was analysed by averaging the data into 45° sectors (Supplementary Discussion and Supplementary Table 5) where the average FWHM is 16° ± 1.8°, consistent with the value of 17.0° ± 0.2° determined when averaging all φ angles. Both analysis methods suggest that the 60% LDH coating film has the highest level of alignment.

### Oxygen barrier performance of flexible barrier film

**Fig. 3:**
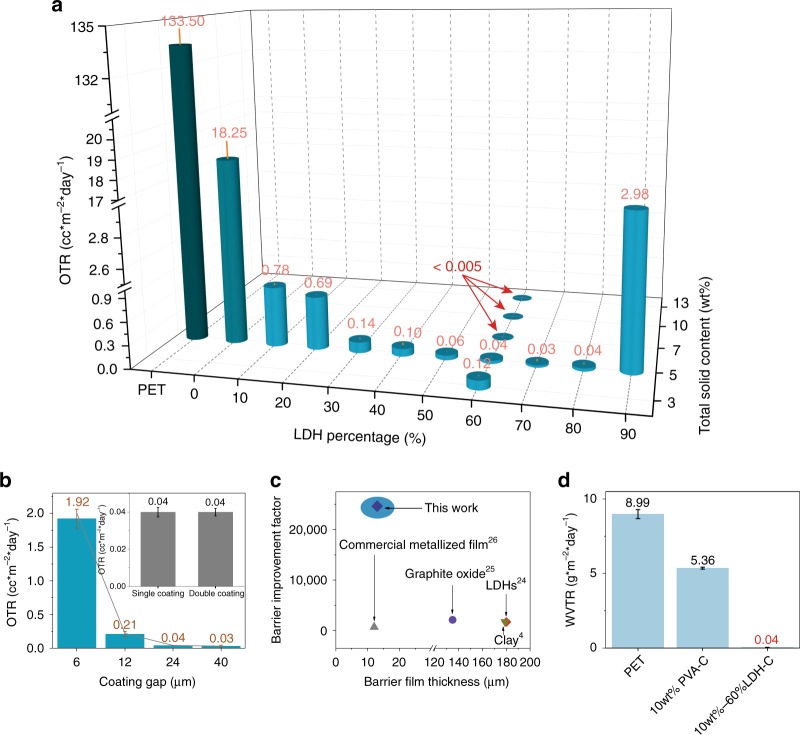
Barrier properties of coated films. **a** OTR plot against LDH% in the coating layer and total solid content of the coating solutions. The films were coated with 24 μm gap. **b** OTR plot against coating gap and the inset shows that the OTR values are very similar by coating a substrate with the same coating layer thickness (a single and double coating process). The grey line is a guide to the eye. The coating solution used to coat all the films contains 3 wt% LDH NS and 2 wt% PVA. **c** Barrier improvement factor plot against barrier film thickness: Comparison of this work and other works with LDH^29^, clay^4^, and graphite oxide^30^ as filler and commercial metallised film^31^. **d** WVTR of the crosslinked barrier film at 10 wt% total solid content where PVA-C and LDH-C indicates that the PVA component was crosslinked by TSMP. PVA MW of 67,000 g mol^−1^ was used to obtain the data in **a**–**c**, PVA MW of 195,000 g mol^−1^ was used to obtain the data in **d**. Error bars represent the standard deviations from more than two measurements. Source data are provided as a Source Data file

Previous reports^21–23^ of gas barrier films based on LDHs have been reported to show increased gas barrier properties. In this study, the oxygen transmission rate (OTR) of the LDH nanosheets coated polymer films were measured at 23 °C, as benchmarks we measured the OTR figures of both an uncoated 12 μm PET film and one coated with 5 wt% aqueous PVA solution give a 0.90 μm dry coating layer (the thickness of all the coating films are listed in Supplementary Table 6, these films displayed OTR values of 133.50 and 18.25 cc m^–2^ per day, respectively (Fig. 3a). Remarkably, the OTR values of the PET films coated with an aqueous PVA solution containing the LDH nanosheets decreased dramatically. For example, using similar coating solution with the same 5 wt% total solid content but comprising 3 wt% LDH nanosheets and 2 wt% PVA produced a PET film with an OTR of 1.92 cc m^−2^ per day (Fig. 3b) at only a 0.09 μm dry coating thickness (Supplementary Fig. 11a). Increasing the dry coating thickness to 0.66 μm decreases the OTR to 0.04 cc m^–2^ per day (Fig. 3b). A single coating process is efficient to decrease the OTR of the coated film to below instrumental detection limits (<0.005 cc m^–2^ per day) (Fig. 3a). The OTR values (Fig. 3b inset) for PET coated once using a 24 μm wet coating gap is very similar to one coated twice with a 12 μm gap which overall produces dry films with similar thickness (*ca*. 0.70 μm) (Supplementary Fig. 10d and Table 6).

We found that the best performing films were observed when the solution contained at least 7 wt% solid content (LDH + PVA) and more specifically when 60–80% of the total solid content in the solution contained LDH nanosheets (Fig. 3a). For example, a solution containing 4.2 wt% LDH nanosheets and 2.8 wt% PVA (7 wt% total solid content) has an OTR below the instrument detection limit of 0.005 cc m^−2^ per day at only a *ca*. 1 μm dry coating thickness (Supplementary Table 6). Interestingly, we found that OTR increased when 90% of the total solid content is LDH nanosheets. We believe this may be as a result of a viscosity change of the coating solution, unlike the other samples, at 90% LDH, the coating solution showed significant shear thinning behaviour (Supplementary Fig. 15). At such a high LDH concentration (90%), the high viscosity of the coating solution means it is difficult for the LDH nanosheets to achieve preferred alignment on the film. Alternatively, the dry coating layer maybe so rigid when the majority of the flexible polymer (PVA) is replaced by LDH that the film became fragile and susceptible to cracking. The OTR of the coated film decreases with increasing total solid content of the solution and the coating gap of the coating bar.

In order to compare oxygen barrier performance between different films with differing thickness coating layers, different barrier materials and different technologies it is useful to determine the O_2_ permeability^24^ (cm^3^(STP) cm cm^−2^ s^−1^ Pa^−1^) and the barrier improvement factor^25^ (BIF) (defined as Ps/Pt, where Ps is the permeability of the substrate and Pt is the permeability of the coated substrate). Our best performing film to date exhibits an O_2_ permeability of at most 0.0007 × 10^−16^ cm^3^ (STP) cm cm^−2^ s^−1^ Pa^−1^ with a BIF of at least 24640 (Supplementary Table 6). As far as we are aware these figures are superior to any existing PET coating technology (Fig. 3c). By comparison, commercially available metallised PET film has an O_2_ permeability and BIF of 0.035 × 10^–16^ cm^3^(STP) cm cm^−2^ s^−1^ Pa^−1^ and 678, respectively (Supplementary Table 6). Currently the best performing oxygen barrier PET film is graphene oxide coated PET with an O_2_ permeability and BIF of 0.0077 × 10^–16^ cm^3^(STP) cm cm^−2^ s^−1^ Pa^−1^ and 2120 respectively (Supplementary Table 6).

Although metallised films are widely used within the food packaging industry due to their high gas barrier properties, they do experience significantly decreased barrier performance after mechanical stress^26^. We used a Gelbo flex tester to simulate the real-life mechanical stresses that food packaging would encounter during manufacturing, handling, and distribution. Under low to very high mechanical stresses, our PET coated films were flexed 50, 100, and 200 times, we find the OTR values remained very similar to that of the film before flexing (Supplementary Fig. 16). The surface of the coated film is smooth (Supplementary Fig. 17a, b) compared to the film coated with control Water-LDH (Supplementary Fig. 18). Even after 200 flex cycles, the surface of the coated film does not show any defects (Supplementary Fig. 17c, d).

### Water vapour barrier (WVTR) performance of barrier film

The water vapour transmission rate (WVTR) of the coated PET film also displayed a significant decrease. We used a high molecular weight PVA (MW 195,000 g mol^−1^) and a food grade crosslinker, trisodium trimetaphosphate (TSMP)^27, 28^, to prepare PET coating film. After crosslinking, PVA becomes insoluble in water, maximising the possibility of preserving the ordered LDH structure when in contact with water vapour. The bare uncoated PET film has a WVTR of 8.99 g m^–2^ per day at 23 °C and 50 % relative humidity. The PET film coated with 10 wt% high MW PVA and crosslinked using TSMP has a WVTR of 5.36 g m^–2^ per day. Replacing 6 wt% of the PVA by the LDH NS (i.e. the coating solution contains 6 wt% LDH + 4 wt% PVA) decreased the WVTR significantly to 0.04 g m^−2^ per day (Fig. 3d).

Our studies suggest that Mg_2_Al-LDH nanosheets prepared by reconstruction in concentrated aqueous glycine forms the basis of a non-toxic, stable coating dispersion when mixed with PVA. Mg_2_Al-LDH nanosheets/PVA coated on 12 μm PET film exhibit O_2_ transmission rates below the detection limit of the instrument (<0.005 cc m^–2^ per day) and a water vapour transmission rate as low as 0.04 g m^−2^ per day, with good mechanical stability, clarity and low haze. The O_2_ permeabilities are at least 50 times lower than the commercial metallised PET film. Furthermore, the absence of a metallic, electrically conducting component within the film structure will offer new applications and recycling opportunities. As a result, we are exploring these LDH nanosheet dispersions as a new barrier technology for flexible food packaging.

### Online content

Any methods, additional references, *Nature Research* reporting summaries, source data, statements of data availability and associated accession codes are available.

## Methods

### Materials

The original platelet Mg_2_Al-LDH sample is commercially available (Alcamizer 1) and was used as purchased from Kisuma Chemicals, Netherlands. Polyvinyl alcohol (PVA) 8–88 (MW: 67,000 g mol^−1^), Poval 56–98 PVA (MW: 195,000 g mol^−1^), Trisodium trimetaphosphate (TSMP) (≥95%), glycine (≥98%), and sodium hydroxide pellets (≥98%) were purchased from Sigma Aldrich. Polyethylene terephthalate (PET) film (12 μm thick) was supplied by SCG chemicals Co. Ltd., Thailand.

### Calcination of LDHs

Typically, the original LDH was calcined at 450 °C for 12 h at a heating rate of 5 °C min^−1^ in air. The calcined LDO was taken out of furnace at ca. 80 °C and stored in a desiccator to avoid slow rehydration in air.

### Reconstruction of LDOs in amino acid solution (LDH NS)

Typically, glycine was mixed with 1 g calcined LDO at 1.5:1 weight ratio in 10 mL water and the mixture was placed in an autoclave and reacted at 100 °C for 48 h to obtain a semi-transparent gel. The obtained gel was then dispersed and stirred in water (usually 100 mL) overnight.

The dispersion is very stable and thus LDH NS are very difficult to collect by centrifuge, even at a high *g* force for a long time. Thus, to improve the yield, the LDH suspension is intentionally precipitated by adding NaOH solutions. Typically, to a solution of 100 mL dispersed gel *ca*. 4–5 mL NaOH (4 M) was added to precipitate the LDH NS. The LDH NS was then collected by centrifuge at 35954* g* force for 10 minutes and washed with D.I. water three times for further characterisations.

After centrifugation, the collected LDH gel was partially dried at 100 °C in oven for 2 h to determine the solid content (the average solid content of three measurements was used in all cases) for coating solution preparation.

### Reconstruction of LDOs in water (Water-LDH)

The LDO was reconstructed under the same conditions as in amino acid solution, except without adding amino acid as a control experiment.

### Coating solution preparation

PVA solution was prepared by dissolving PVA resin in water at ca. 90 °C under reflux for an hour. 10 wt% PVA stock solution was used to prepare coating solution. Reconstructed LDH gel was mixed and stirred overnight with 10 wt% PVA solution and water to make coating solution with different total solid contents and LDH loadings. The coating solutions typically contain 95 wt% water and 5 wt% solid where LDH is 3 wt% and PVA is 2 wt%.

### Coating process

PET substrate was coated with the coating solutions by a semi-automatic coater (K control coater, RK PrintCoat instruments Ltd, UK) at a coating speed equivalent to 10.67 m min^–1^. After coating, the PET films are dried at room temperature for about 1 h before testing.

### Crosslinking of PVA for WVTR

PVA with molecular weight of 195,000 g mol^−1^ was only used to improve water vapour barrier of the coated film. Trisodium trimetaphosphate (TSMP) was used to crosslink PVA following a previous report^27^. Typically, 5 g of 10 wt% PVA solution (or LDH/PVA mixture) was mixed with 0.08 mL of 0.16 M TSMP and 0.03 mL of 2.5 M NaOH right before coating. After coating, the coated film was dried and cured at 100 °C for 5 h.

### OTR testing

The OTR of the barrier films were tested on M8001 oxygen permeation analyser (Systech Instruments, UK) at 23 °C and zero relative humidity. The instrument testing limit is 0.005 cc m^−2^ per day. The testing complies with ASTM D-3985.

### WVTR testing

The WVTR of the barrier films were tested on M7001 water vapour permeation analyser (Systech Instruments, UK) at 23 °C and 50% relative humidity. The testing complies with ASTM standard F-1249.

### XRD measurements

The samples for XRD measurements of LDOs reconstructed in glycine were prepared by quenching the reaction by liquid nitrogen after certain periods of time (from 1 minute to 48 h) to rapidly cool down the temperature. After the reaction mixture temperature rose back to room temperature, the mixture was put into an aluminium holder and covered with Mylar® film (0.25 mil, XRF Window Film, Fisher Scientific) to avoid drying of the samples. The samples were scanned at a canning speed of 0.04° min^–1^.

The barrier films were taped on to an aluminium holder to make XRD measurements with the coated side facing the incident X-ray beam. All XRD measurements were recorded on Bruker D8 diffractometer (40 kV and 30 mA) with Cu Kα radiation (*λ*_1_ = 1.544 Å and *λ*_2_ = 1.541 Å).

### Estimation of crystallite sizes

Scherrer equation is used to estimate the size of crystallites which correlates to the peak broadening in an X-ray diffraction pattern.

2 $$D = \frac{{k\lambda }}{{\beta {\rm{cos}}\theta }},$$

where D is the mean size of crystallites perpendicular to the diffraction plane; *k* is a dimensionless shape factor (usually is 0.89 for LDHs); λ is the wavelength of the X-ray (λ = 0.15425 nm); β is the peak broadening at half maximum intensity (FWHM) after subtracting the instrument line broadening in radian; *θ* is the Bragg angle.

### FT-IR measurements

IR spectra were recorded on a Varian FTS-7000 Fourier transform infra-red spectrometer fitted with a DuraSamplIR Diamond ATR. The samples were prepared as described in XRD measurements and tested as it is.

### TGA measurements of LDHs and barrier films

The measurements were recorded on a Mettler Toledo TGA/DSC 1 system from 30 to 600 °C (film samples) and from 30 to 800 °C (LDHs samples) at a heating rate of 10 °C min^−1^ under N_2_ atmosphere.

### TEM measurements of LDHs and cross-sectional TEM sample preparation

All TEM images were obtained on a JEOL JEM-2100 transmission electron microscope with an accelerating voltage of 200 kV. The coated PET films were first embedded into epoxy, and slices of ca. 80–100 nm thickness were cut on a Reichert-Jung Ultracut E ultramicrotome from the embedded epoxy sample. The slices were deposited on 75-mesh copper grids for imaging.

### Viscosity measurements

Dynamic viscosity was measured on a HR-2 discovery hybrid rheometer (TA instruments) using 60 mm aluminium cone plate with an angle of 1.01° and a truncation gap of 30 μm at 25 °C.

### SEM imaging

SEM images were taken on a Zeiss Merlin-EBSD scanning electron microscope with an operating voltage of 5 kV. The films were first coated with ca. 10 nm gold before imaging.

### AFM measurements

The coating layer thickness and thickness of LDHs were measured by a NanoScope MultiMode atomic force microscope using tapping mode with a silicon tip coated with aluminium with a force constant of 40 N.m^–1^. LDHs samples were diluted into *ca*. 0.01 mM and spin coated on freshly cleaved mica wafer for AFM imaging.

### Mechanical flex of the films

The films were conditioned at 23 ± 2 °C and 50 ± 5% RH for 48 h before the flex. All films were flexed by a Gelbo flex tester (IDM instruments) following ASTM F392-93 standard.

### Optical measurements of the barrier films

Haze and transparency of the films were tested by a haze-gard I haze meter (BYK instruments) following ASTM D1003-00 Standard test method. The film samples were conditioned at 23 ± 2 °C and 50 ± 5% RH for 48 h before testing.

### Pole figure measurements

For Pole figure measurements a Panalytical X’Pert Pro MRD was used. This is equipped with a 4-bounce Ge Hybrid Monochromator giving pure Cu Kα_1_ radiation and a Pixcel detector as a point detector with an 8.5 mm active length. This provides each pole figure with a *2θ* range of 1.5°, allowing us to isolate the scattering from the PVA/LDH phase and LDH/Glycine phase scattering. The samples containing 20, 60, and 90% LDH in the coating layer were mounted on a glass slide using double-sided tape and oriented so that at *φ* = 0° the top of the sample.

The pole figure measurement consists of a series of *φ* scans (rotation of the sample about the surface normal) made at a number of different *ψ* angles (sample tilt angle). Each *φ* scan was from 0 to 360° with a 2° step size and a counting time of 0.88 s per position. A phi scan was made every 2° from 0 to 26° in *ψ* giving a total collection time per pole figure of 45 min. For each sample a measurement was made with the detector fixed at 8.5° and 11.5° in *2θ* to ensure the diffracted intensity was from the PVA/LDH phase and LDH/Glycine phase, respectively.

**Degree of orientation**

3 $$\partial = \frac{{180 - {\mathrm{FWHM}}}}{{180}} \times 100,$$

where FWHM is the full width at half maximum obtained by pole figure measurements.

### Barrier improvement factor

Barrier improvement factor (BIF) is defined as Ps/Pt, where Ps is the permeability of the substrate and Pt is the permeability of the coated substrate.

## Supplementary information

Supplementary Information

Source Data

## Data Availability

The data that support the findings of this study are available from the corresponding author upon reasonable request.

## References

[1] Coleman JN (2011). Two-dimensional nanosheets produced by liquid exfoliation of layered materials. Science.

[2] Yu J, Wang Q, O’Hare D, Sun L (2017). Preparation of two dimensional layered double hydroxide nanosheets and their applications. Chem. Soc. Rev..

[3] Ding F (2017). Biomimetic nanocoatings with exceptional mechanical, barrier, and flame-retardant properties from large-scale one-step coassembly. Sci. Adv..

[4] Priolo MA, Gamboa D, Holder KM, Grunlan JC (2010). Super gas barrier of transparent polymer− clay multilayer ultrathin films. Nano Lett..

[5] Das P (2015). Nacre-mimetics with synthetic nanoclays up to ultrahigh aspect ratios. Nat. Commun..

[6] MgAl-Layered Double Hydroxides have EU Approval for Food Contact (PM/REF: 34690 & 60080, http://www.legislation.gov.uk/uksi/2006/1401/pdfs/uksi_20061401_en.pdf) and US FDA registration as GRAS (UNII: 17432CG1KU, https://drugs.ncats.io/drug/17432CG1KU).

[7] Liu Z (2006). Synthesis, anion exchange, and delamination of Co− Al layered double hydroxide: assembly of the exfoliated nanosheet/polyanion composite films and magneto-optical studies. J. Am. Chem. Soc..

[8] Hu G, Wang N, O’Hare D, Davis J (2007). Synthesis of magnesium aluminium layered double hydroxides in reverse microemulsions. J. Mater. Chem..

[9] Hibino T (2004). Delamination of layered double hydroxides containing amino acids. Chem. Mater..

[10] Wu L, Hu Z, Chen G, Li Z (2015). A convenient green preparation of layered double hydroxide/polyacrylamide nanocomposite hydrogels with ultrahigh deformability. Soft Matter.

[11] Cavani F, Trifirò F, Vaccari A (1991). Hydrotalcite-type anionic clays: Preparation, properties and applications. Catal. Today.

[12] Wyman J (1936). The dielectric constant of solutions of dipolar ions. Chem. Rev..

[13] Millange F, Walton RI, O’Hare D (2000). Time-resolved in situ X-ray diffraction study of the liquid-phase reconstruction of Mg–Al–carbonate hydrotalcite-like compounds. J. Mater. Chem..

[14] Cussler E, Hughes SE, Ward WJ, Aris R (1988). Barrier membranes. J. Memb. Sci..

[15] Choudalakis G, Gotsis A (2009). Permeability of polymer/clay nanocomposites: a review. Eur. Polym. J..

[16] Nair R, Wu H, Jayaram P, Grigorieva I, Geim A (2012). Unimpeded permeation of water through helium-leak–tight graphene-based membranes. Science.

[17] Li P (2015). Highly effective anti-corrosion epoxy spray coatings containing self-assembled clay in smectic order. J. Mater. Chem. A.

[18] Cao Y, Irwin PC, Younsi K (2004). The future of nanodielectrics in the electrical power industry. IEEE Trans. Dielect. Elect. Insul.

[19] Xu K, Chen G, Shen J (2013). Exfoliation and dispersion of micrometer-sized LDH particles in poly(ethylene terephthalate) and their nanocomposite thermal stability. Appl. Clay Sci..

[20] Zhu B (2015). Hierarchical nacre mimetics with synergistic mechanical properties by control of molecular interactions in self‐healing polymers. Angew. Chem. Int. Ed..

[21] Yu, J. *et**al*. Synthesis of Layered Double Hydroxide Single-Layer Nanosheets in Formamide. *Inorg*. *Chem*. **55**, 12036–12041 (2016). 10.1021/acs.inorgchem.6b02203 27802040

[22] Xie J (2018). Scale-up fabrication of biodegradable poly(butylene adipate-co-terephthalate)/organophilic–clay nanocomposite films for potential packaging applications. ACS Omega.

[23] Carvalho HWP, Leroux F, Briois V, Santilli CV, Pulcinelli SH (2018). Thermal stability of PMMA–LDH nanocomposites: decoupling the physical barrier, radical trapping, and charring contributions using XAS/WAXS/Raman time-resolved experiments. RSC Adv..

[24] Roberts A (2002). Gas permeation in silicon-oxide/polymer (SiOx/PET) barrier films: role of the oxide lattice, nano-defects and macro-defects. J. Memb. Sci..

[25] Priolo MA, Holder KM, Gamboa D, Grunlan JC (2011). Influence of clay concentration on the gas barrier of clay–polymer nanobrick wall thin film assemblies. Langmuir.

[26] Parkar, A. *Effect of Flexing on the Barrier Properties of Metallized Films*. MSc thesis, Rochester Institute of Technology (2005).

[27] Chaouat M (2008). A novel cross‐linked poly (vinyl alcohol)(PVA) for vascular grafts. Adv. Funct. Mater..

[28] Lack S, Dulong V, Picton L, Le Cerf D, Condamine E (2007). High-resolution nuclear magnetic resonance spectroscopy studies of polysaccharides crosslinked by sodium trimetaphosphate: a proposal for the reaction mechanism. Carbohydr. Res..

[29] Dou Y (2015). Transparent, ultrahigh‐gas‐barrier films with a brick–mortar–sand structure. Angew. Chem. Int. Ed..

[30] Chen JT (2014). Enhancing polymer/graphene oxide gas barrier film properties by introducing new crystals. Carbon.

[31] Jamieson E, Windle A (1983). Structure and oxygen-barrier properties of metallized polymer film. J. Mater. Sci..

