# Supplementary material for: High gas barrier coating using non-toxic nanosheet dispersions for flexible food packaging film
Source: Nat Commun. 2019 Jun 11;10:2398. doi: 10.1038/s41467-019-10362-2 (PMC6560082; doi:10.1038/s41467-019-10362-2)
Supplement: Supplementary file 1 — Supplementary Information [file 41467_2019_10362_MOESM1_ESM.docx]

**Supplementary Information**

**High gas barrier coating using non-toxic nanosheet dispersions for flexible food packaging film**

Yu et al.


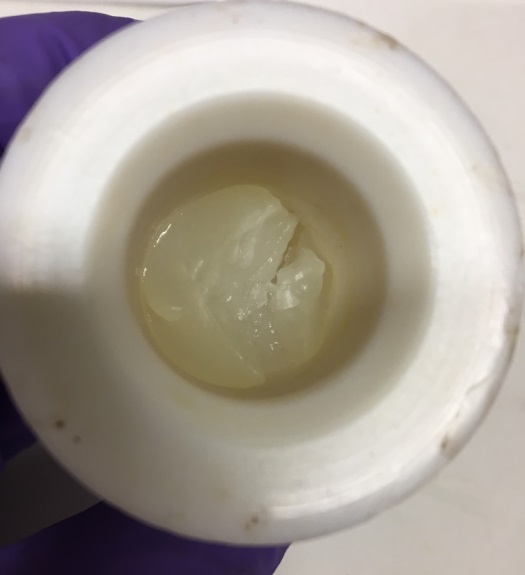


**Supplementary Fig. 1** Digital image of the reconstructed LDH gel.

**Supplementary Table 1** Dielectric constant of selected amino acids and peptides.

| Amino acids | Structure | Solubility in water (*M*) | Dielectric constant  (25 °C, 2 M) |
| --- | --- | --- | --- |
| Glycine |  | 3.329 | 125.2^1^ |

**Supplementary Table 2** Mg/Al ratios of the original LDH, LDH reconstructed in glycine (LDH NS) and control LDH reconstructed in water (Water-LDH): the metal ratios stay very close to each other indicating that the reconstruction process does not change the metal ratio.

| Samples | Average Mg/Al molar ratio |
| --- | --- |
| Original LDH | 2.11 ± 0.01 |
|  |  |
|  |  |
| LDH NS | 2.13 ± 0.01 |
|  |  |
|  |  |
| Water-LDH | 2.10 ± 0.01 |
|  |  |
|  |  |

**
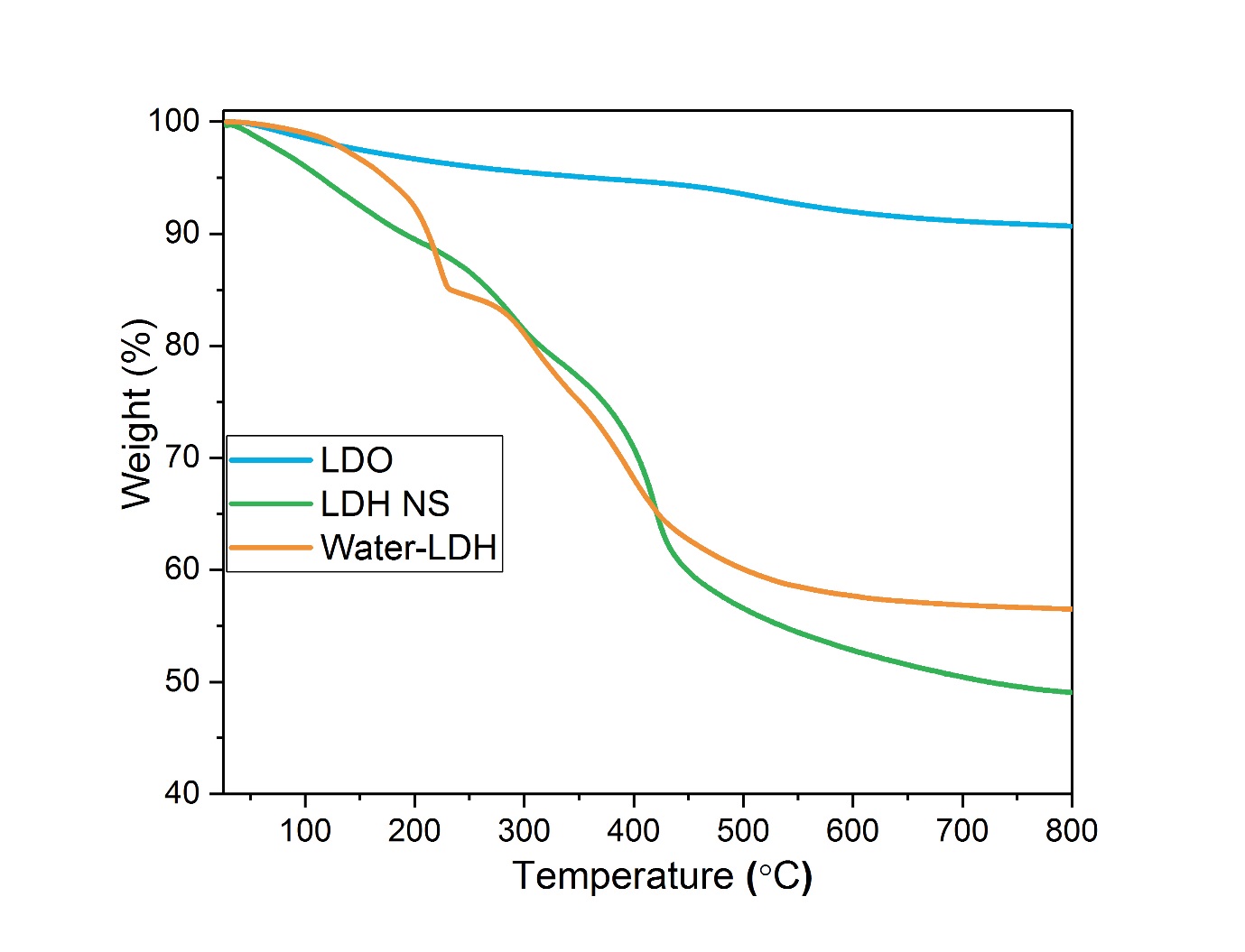
**

**Supplementary Fig. 2** Thermal gravimetric analysis of LDO calcined at 450 °C, LDH NS reconstructed in glycine and LDH reconstructed in water (Water-LDH).

**Supplementary Table 3** Glycine content in LDH NS calculated from TGA.

| Samples | Weight loss at 200 °C (wt%) | Total weight loss at 800 °C (wt%) | Glycine content (wt%)  (weight loss difference at 800 °C) |
| --- | --- | --- | --- |
| LDO | 3.32 | 9.33 |  |
| LDH NS | 10.5 | 50.94 | 7.44 |
| Water-LDH | 7.63 | 43.5 |  |

**
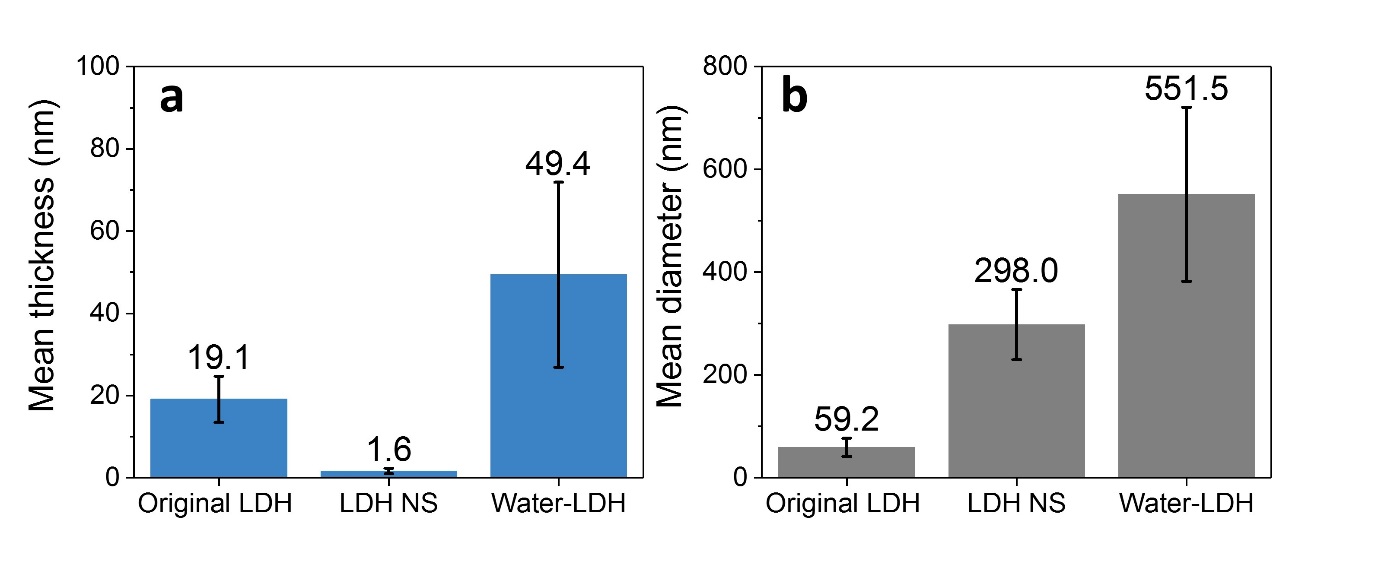
**

**Supplementary Fig. 3** Mean thickness (a) and diameter (b) of original LDH, LDH reconstructed in glycine (LDH NS) and control LDH reconstructed in water (Water-LDH). Thickness and diameter are obtained from AFM measurements of samples at more than three different spots. Aspect ratio was calculated by diameter divided by thickness of individual particles. Error bars represent the standard deviations of more than 30 measurements. Source data are provided as a Source Data file.

**
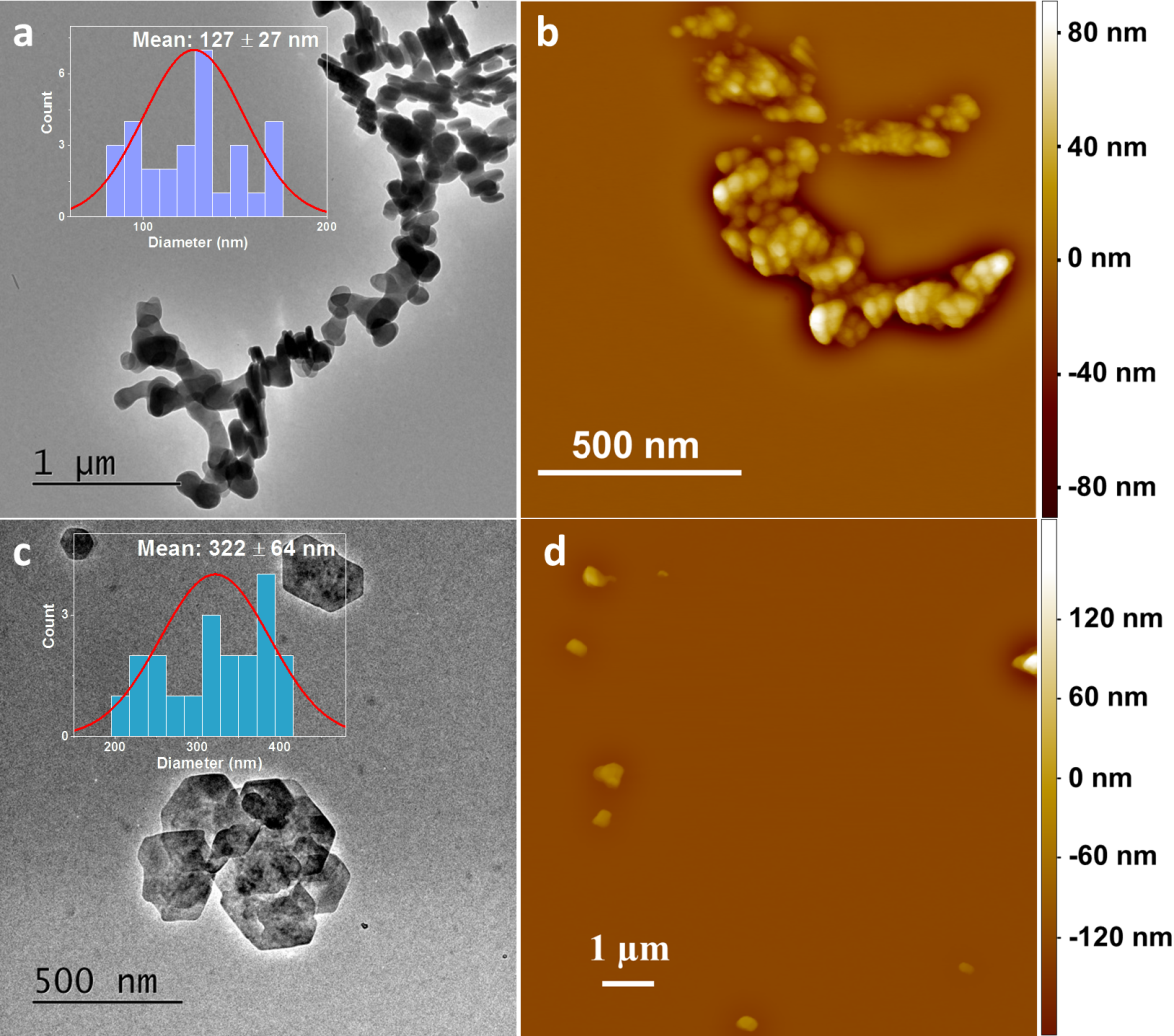
**

**Supplementary Fig. 4** Particle size of LDHs: TEM and AFM images of the original LDH (a and b) and the control LDH reconstructed in water (c and d) (inset in TEM images represents the diameters measured by TEM).


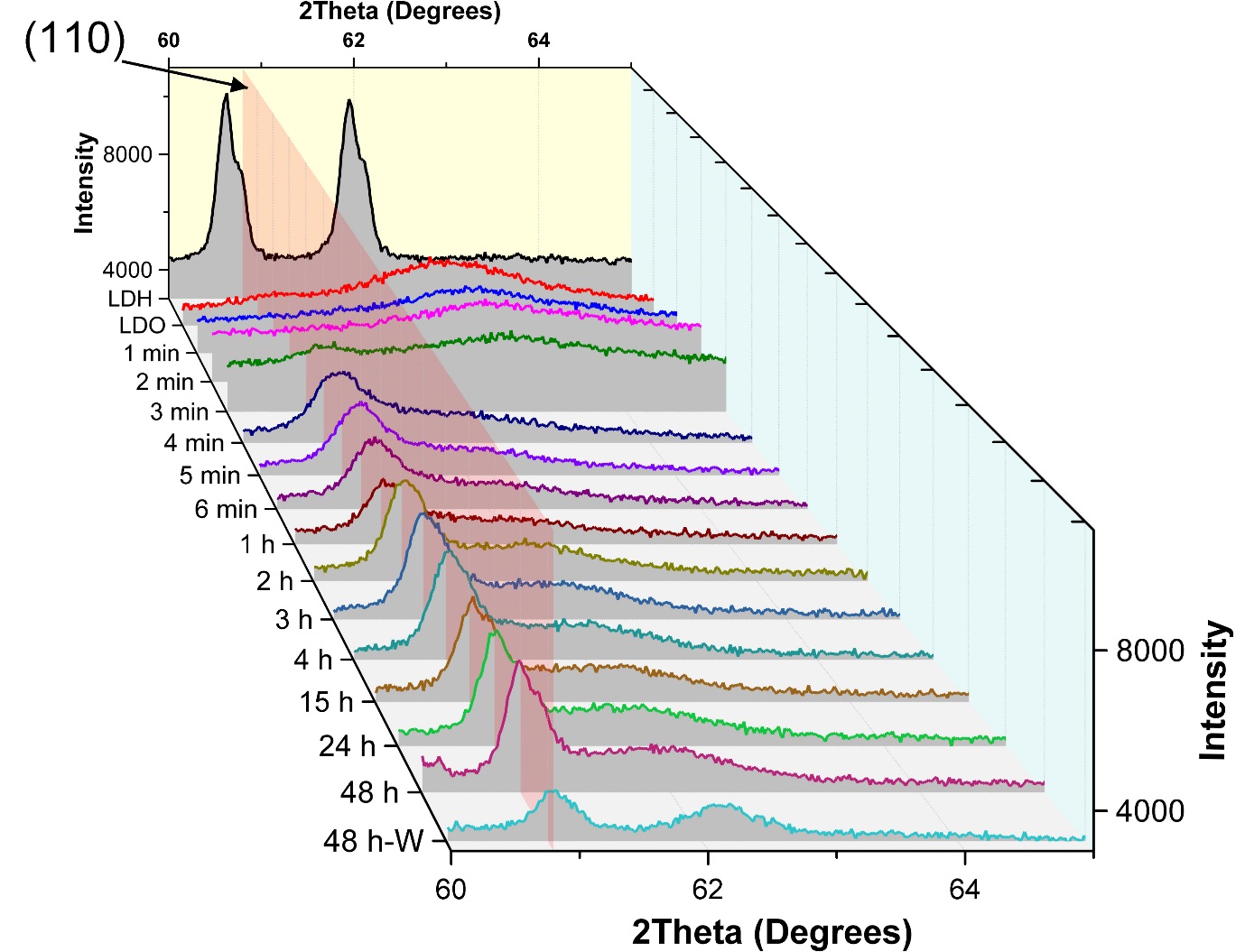


**Supplementary Fig. 5** XRD patterns of MgAl-LDH NS as function of reconstruction time (1 min- to 48 hours): platelet diameter monitored by observing the in-plane 110 Bragg reflection of the LDH (48 h-W, W represent washed sample) compared with the original LDH, calcined LDO and washed 48 h sample.





**Supplementary Fig. 6** XRD patterns of MgAl-LDH NS at reconstruction time varied from 1 minute to 48 hours: thickness growth monitored by observation of the 003 Bragg reflection of LDH. (48 h-W, W represent washed sample) compared with the original LDH, calcined LDO and washed 48 h sample.

**
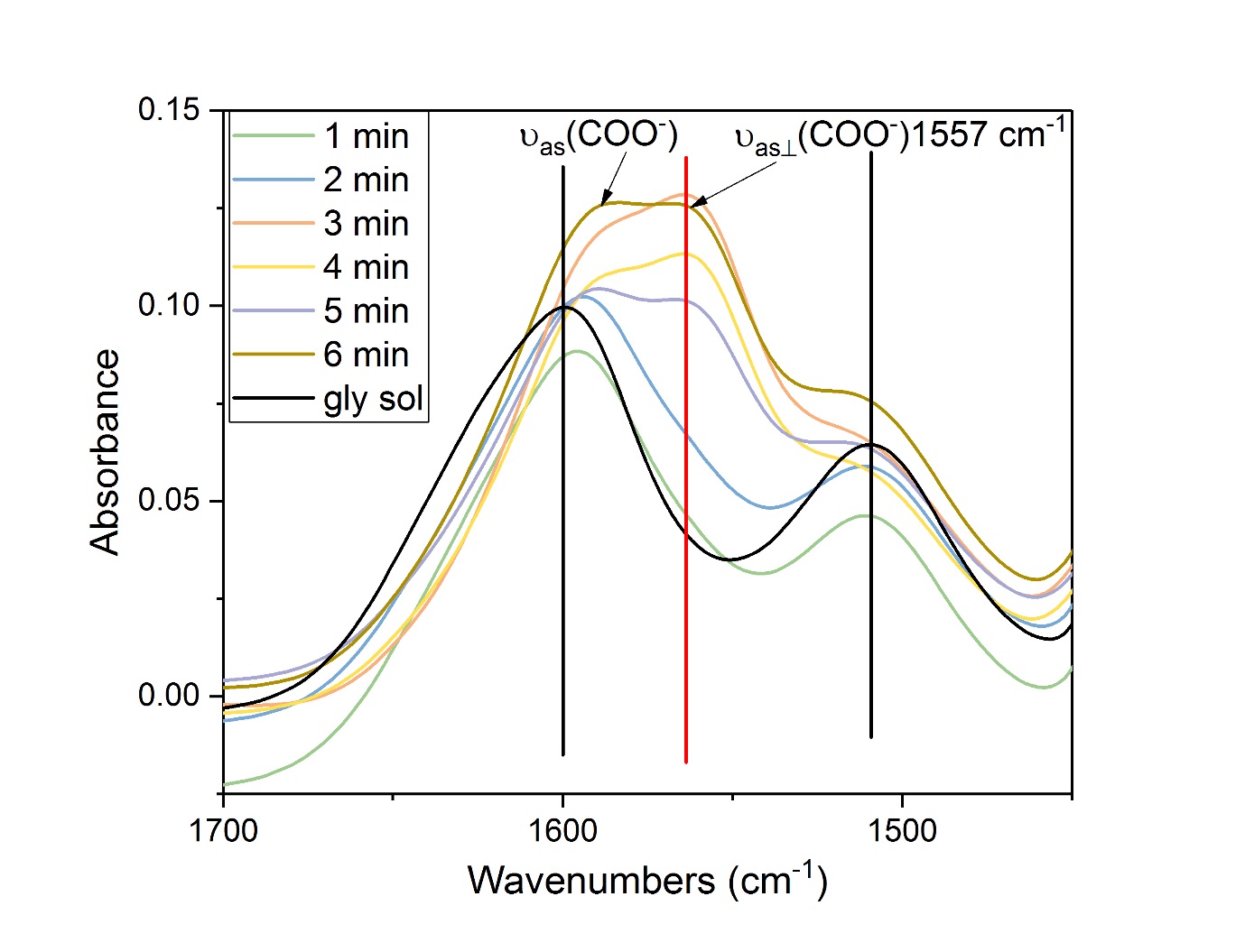
**

**Supplementary Fig. 7** IR spectra of LDH reconstructed in glycine solution for various periods of time.


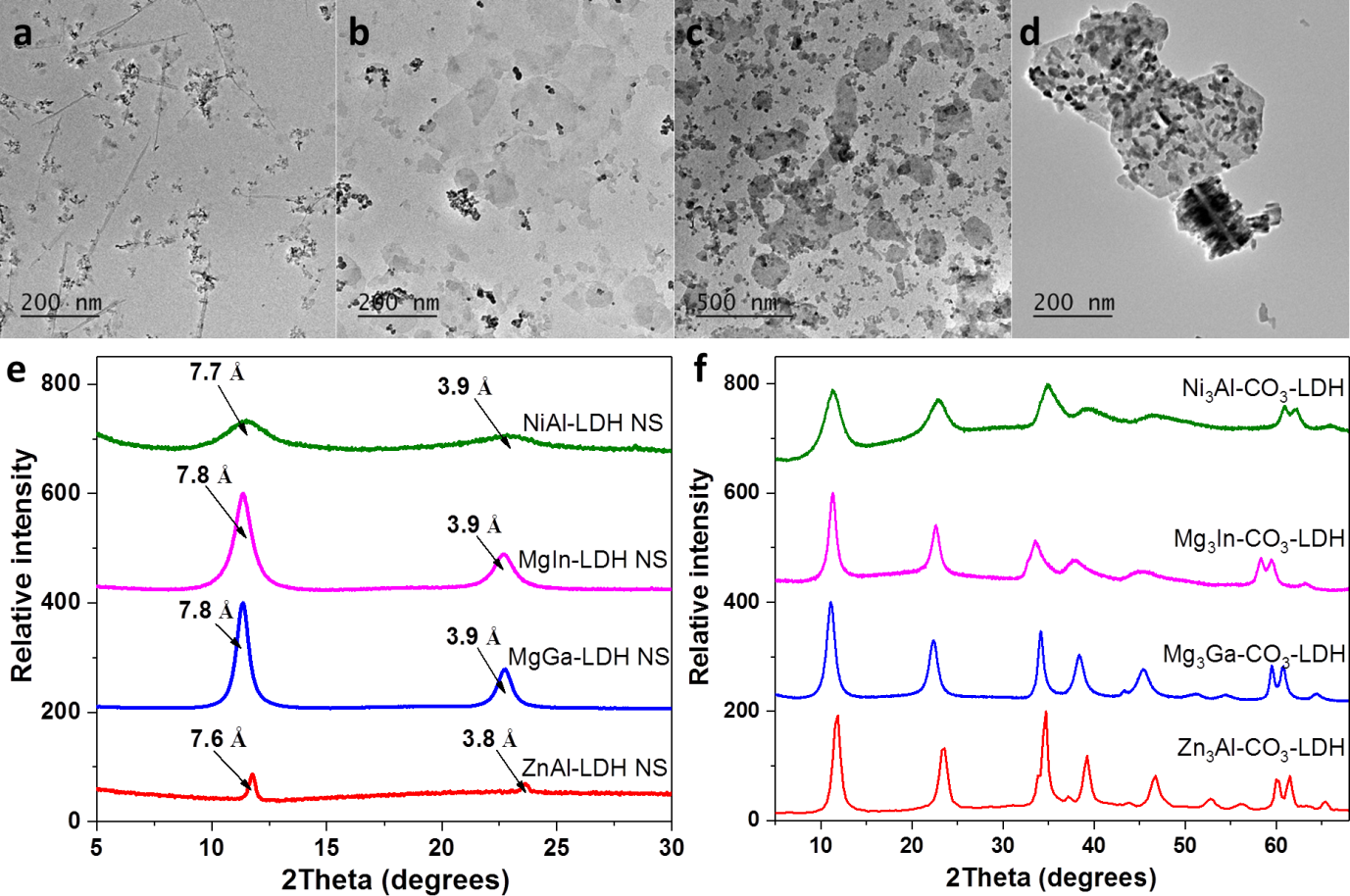


**Supplementary Fig. 8** Reconstruction of other LDHs containing various metal cations. TEM images of reconstructed NiAl (**a**), MgIn (**b**), MgGa (**c**), and ZnAl-LDH NS (**d**); XRD patterns of the reconstructed LDHs NS (**e**) and original LDHs (**f**).


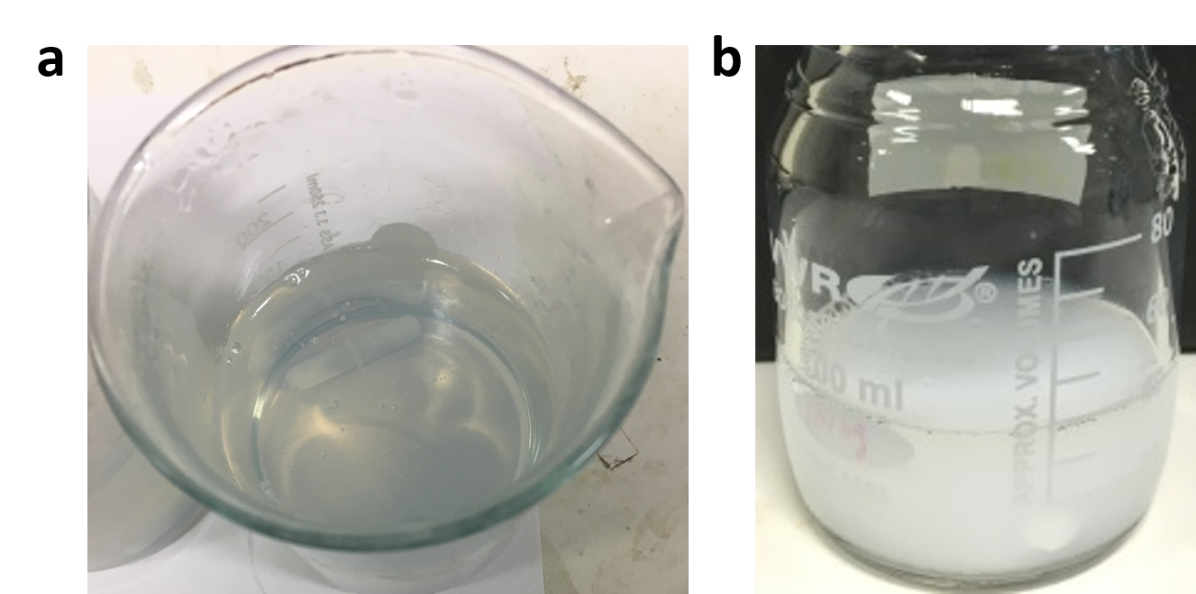


**Supplementary Fig. 9** Digital images of LDH NS dispersion in water (**a**) and stable LDH/PVA coating solution (**b**).


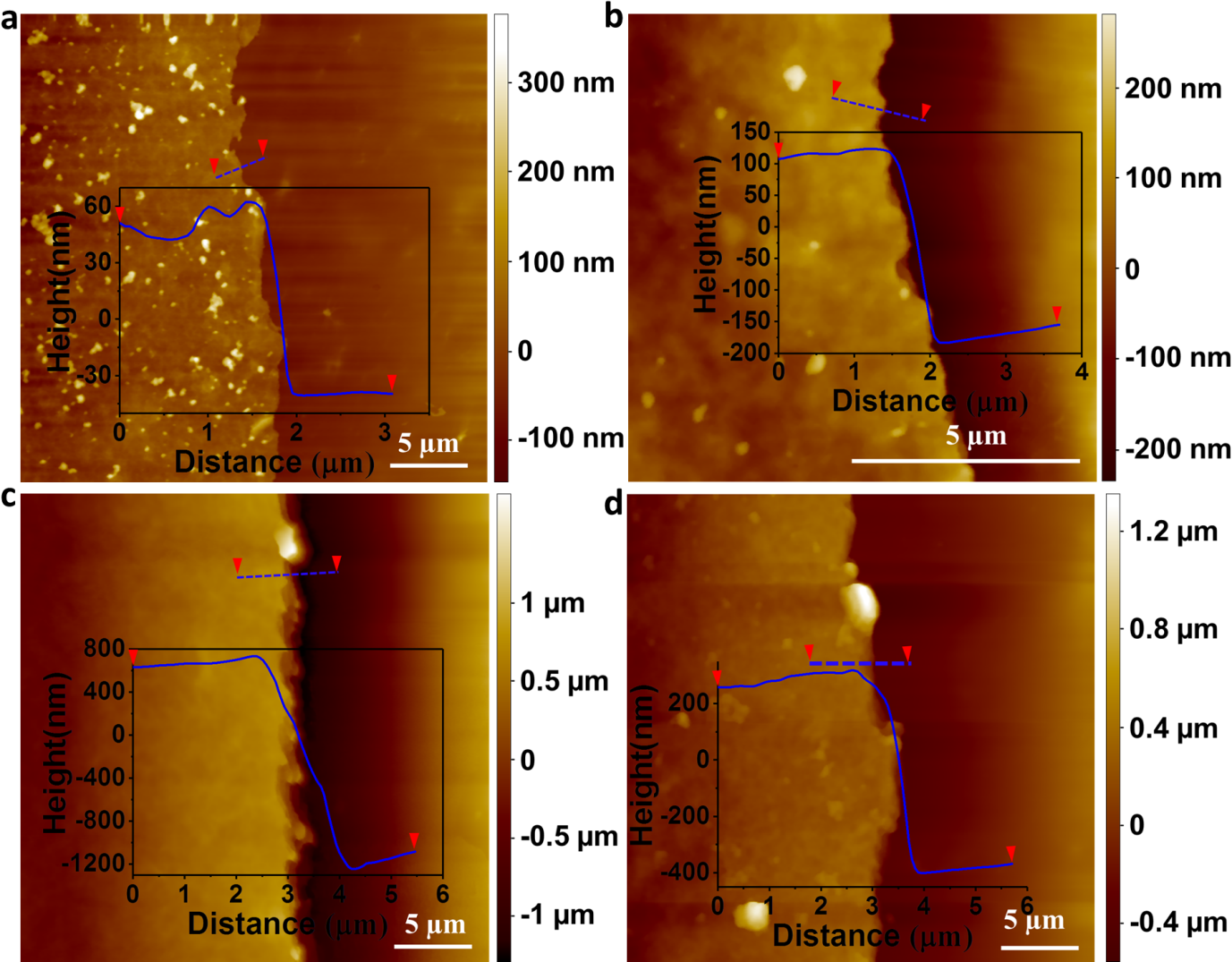


**Supplementary Fig. 10** Thickness of coating layers measured by AFM. Coating layer thickness of film coated with 6 (**a**), 12 (**b**), and 40 μm (**c**) coating gap and films coated twice with 12 μm (**d**) coating gap which is very close to the thickness of film coated with 24 μm coating gap (Coating solution is 5wt%-60% LDH and PVA with MW of 67,000 was used for all the coating films).


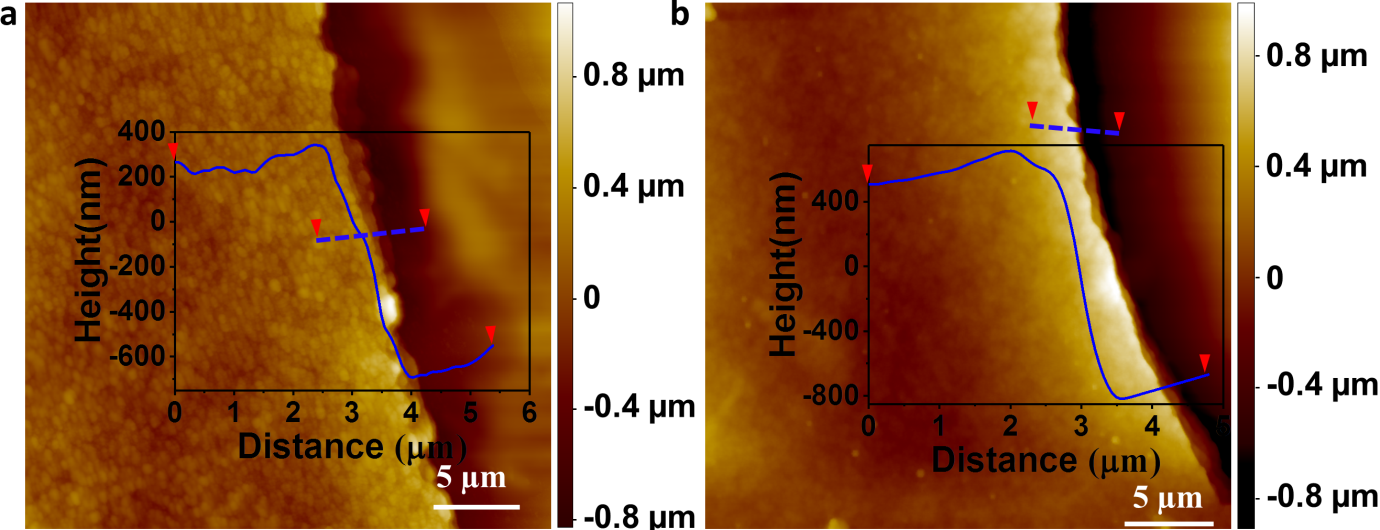


**Supplementary Fig. 11** Thickness of coating layers measured by AFM. Coating layer thickness of film coated with 80% LDH in 5 wt% total solid content solution (**a**) and with 60% LDH in 10 wt% total solid content solution (**b**). The coating gap of the rod is 24 μm. PVA with MW of 67,000 was used for all the coating films.


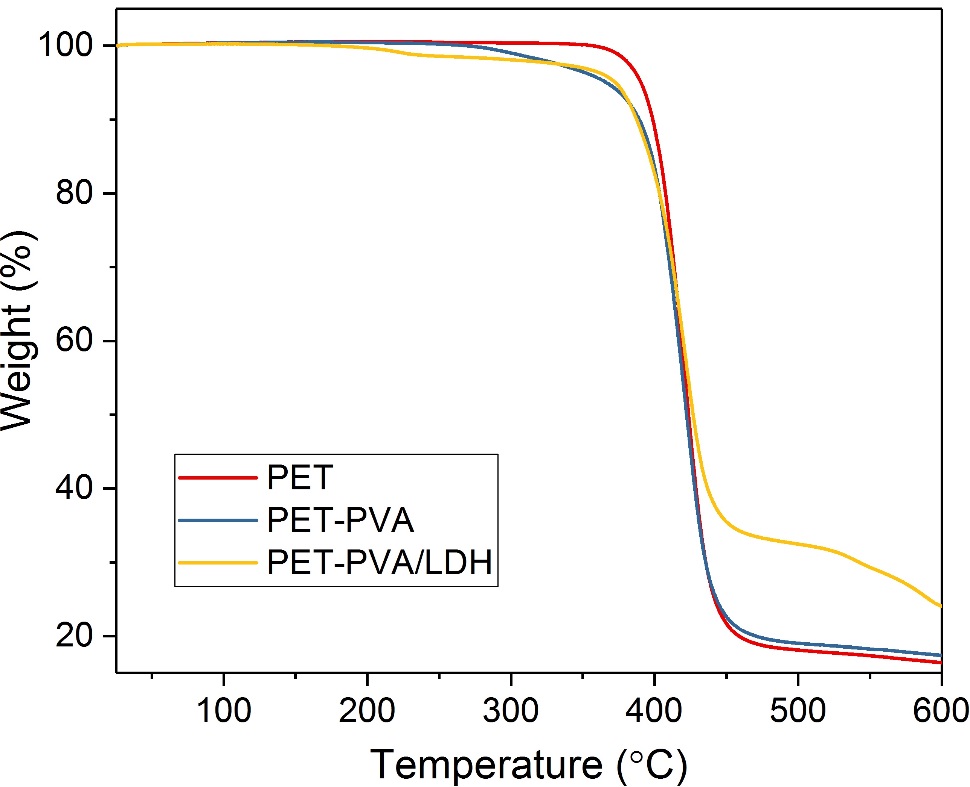


**Supplementary Fig. 12** Thermal properties of the barrier films. Thermogravimetric analysis curves of PET film, PET film coated with PVA and PVA/LDH NS. (PVA MW of 67,000 g/mol was used and the coating solution contains 7 wt% PVA for PVA coated PET film, and 4.2 wt% LDH NS + 2.8 wt% PVA for PVA/LDH coated PET film).

**Supplementary Table 4.** Thermal stabilities of PET film, PET film coated with PVA and PVA/LDH NS. **(**PVA MW of 67,000 g/mol was used and the coating solution contains 7 wt% PVA for PVA coated PET film, and 4.2 wt% LDH NS + 2.8 wt% PVA for PVA/LDH coated PET film).

| Sample | T_max_ (°C) | Residue at 600 °C (%) |
| --- | --- | --- |
| PET | 421.3 | 16.3 |
| PET-PVA | 420.8 | 17.3 |
| PET-PVA/LDH | 421.1 | 23.6 |


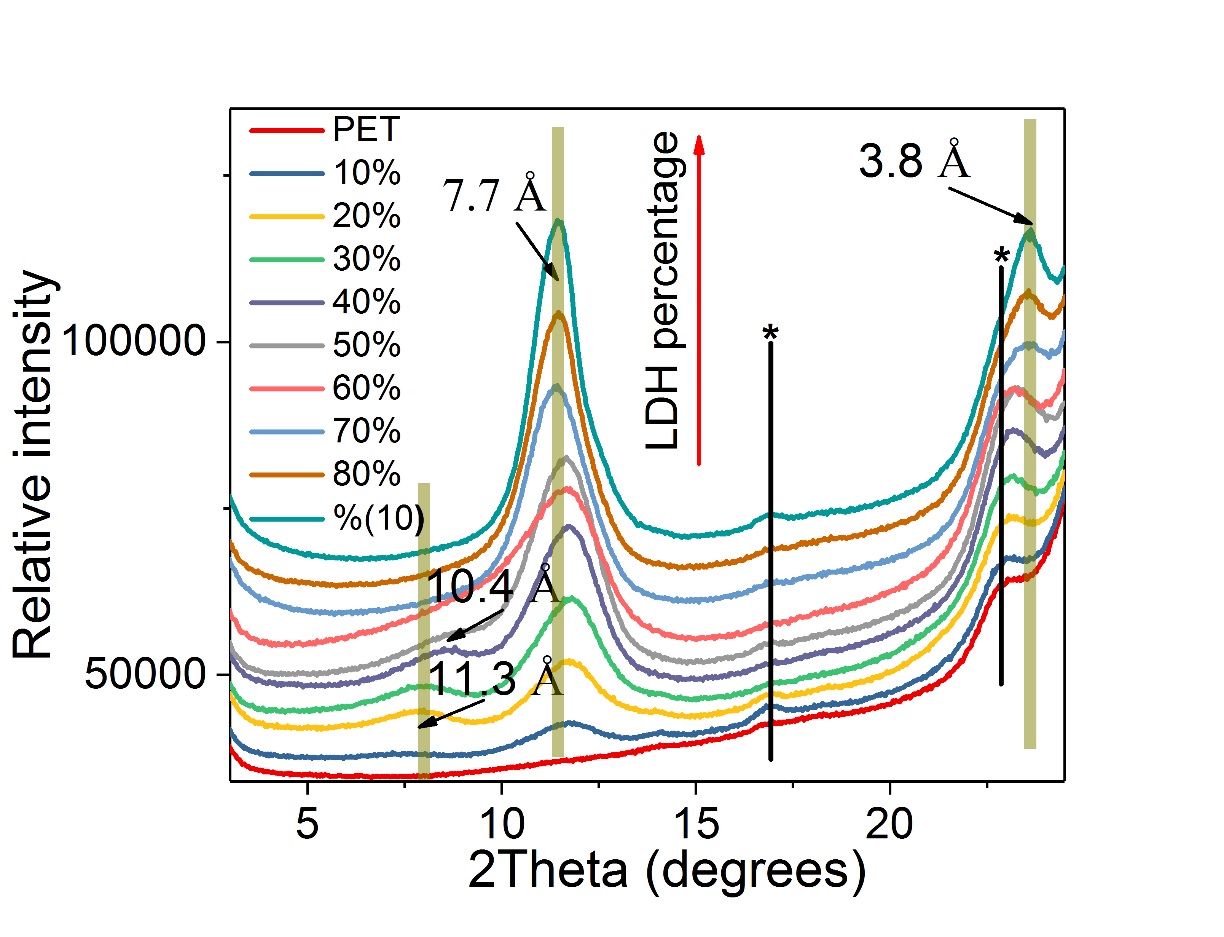


**Supplementary Fig. 13** XRD measurements of barrier films containing 10-90% LDH in the coating layer (* indicates diffractions from PET substrates). The total solid content of each coating solution is 5 wt% and all the films discussed in this figure were coated with 24 μm coating gap; PVA with MW of 67,000 was used for all the film samples.


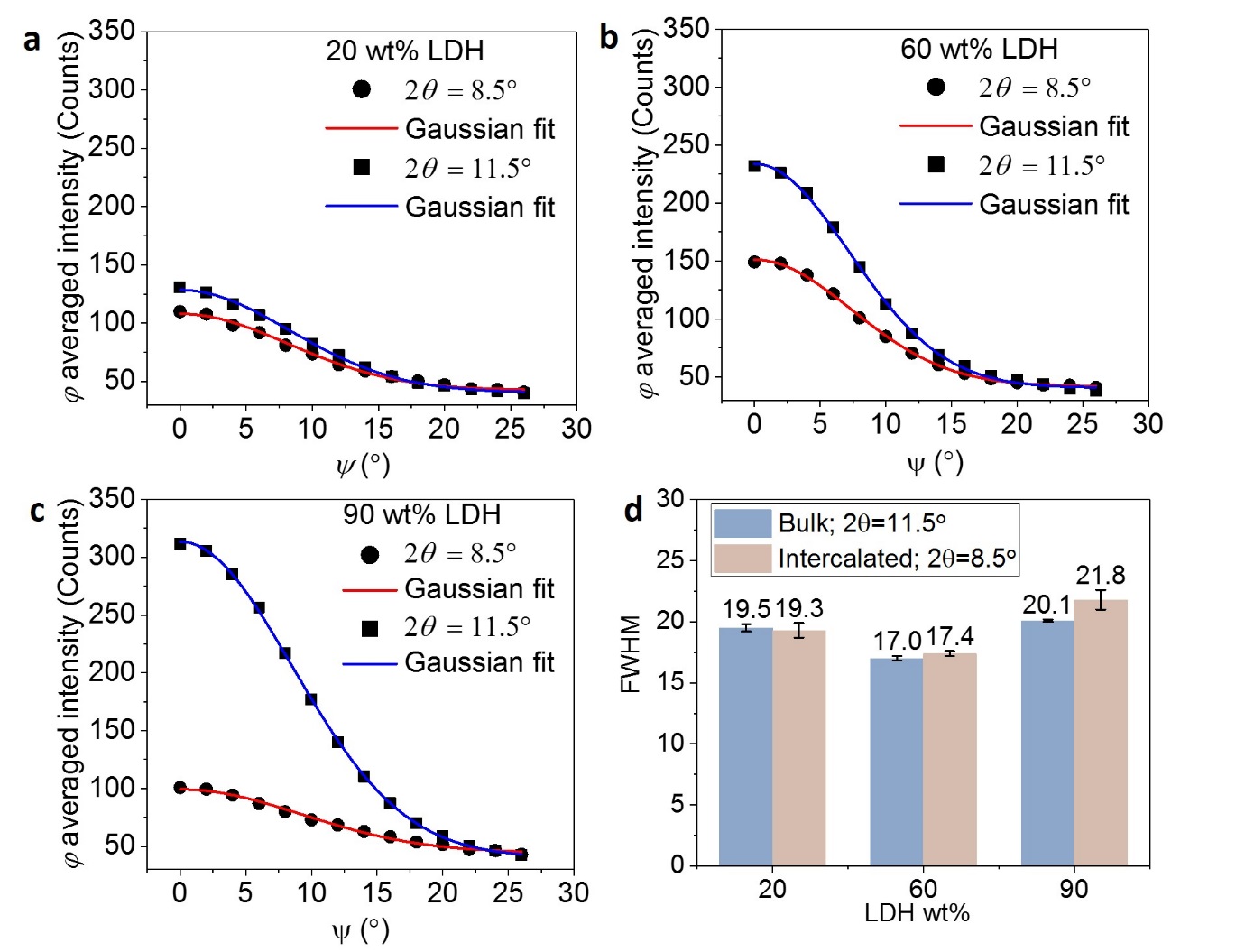


**Supplementary Fig. 14** Orientation of LDH NS in barrier films. The *φ* averaged intensity plotted against the ψ angle for coating film containing 20% (**a**), 60% (**b**) and 90% LDH (**c**). Data measured at a *2θ* of 8.5° (LDH/PVA phase) are marked by the black circles and fitted with a Gaussian coloured red and data measured at a *2θ* of 11.5° (LDH/Glycine phase) are marked by black squares and fitted with a blue Gaussian. **d**, FWHMs in degrees plotted against LDH% in coating layer (The total solid content of each coating solution is 5 wt% and PVA with MW of 67,000 was used for all the coated film samples discussed in this figure). Error bar represents standard error of curve fitting. Source data are provided as a Source Data file.

**Supplementary Discussion**

For the coating film containing 60% LDH, the pole figure measured at *2θ*=8.5° was analysed by averaging the data into 45° sectors. The eight separate data sets were then fitted with Gaussians but this time allowing the peak centre to also refine. The FWHM and peak centres from these fits are included in Table S4. The results show there is a small variation in the FWHM depending on the in-plane orientation. The average FWHM of 16(2)° is consistent with the value of 17.0(2)° determined when averaging all *φ* angles. This suggests either method will give a reasonable value for the FWHM of LDH layer orientation.

**Supplementary Table 5** FWHM and peak centres of Gaussian fits for *φ* sector data measured on coating film with 60% LDH at *2θ*=8.5°.

|  | *φ* Sector (°) | | | | | | | |  | |  | |
| --- | --- | --- | --- | --- | --- | --- | --- | --- | --- | --- | --- | --- |
|  | 0-45 | 45-90 | 90-135 | 135-180 | 180-215 | 215-270 | 270-315 | 315-360 | | Average (°) | | STD (°) |
| FWHM (°) | 14.9 | 17.0 | 18.7 | 13.1 | 16.4 | 17.6 | 16.7 | 13.7 | | 16.0 | | 1.8 |
| Uncertainty (°) | 1.1 | 0.7 | 0.3 | 0.4 | 0.8 | 0.8 | 1.1 | 0.5 | |  | |  |
|  |  |  |  |  |  |  |  |  | |  | |  |
| Xc (°) | -0.5 | 0.0 | 4.0 | 1.3 | -3.6 | -2.5 | 3.0 | 2.4 | | 0.5 | |  |
| Uncertainty (°) | 0.7 | 0.4 | 0.1 | 0.2 | 0.7 | 0.6 | 0.4 | 0.2 | |  | |  |


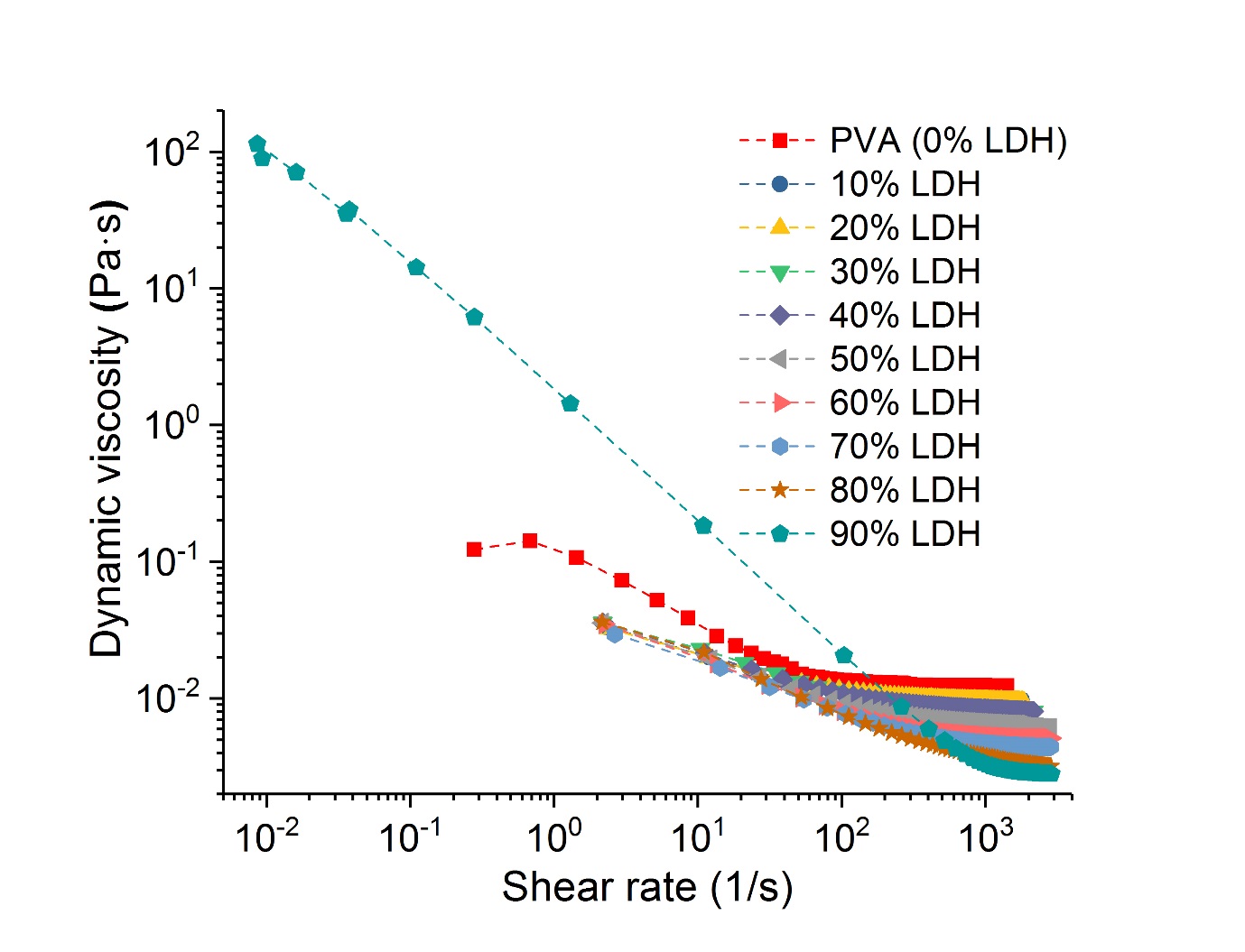


**Supplementary Fig. 15** Dynamic viscosity of the coating solution with LDH percentage varied from 10 to 90% compared with control PVA solution which, unlike the rest of the samples, the 90% LDH sample showed significant shear thinning effect (the total solid content of each coating solution is 5 wt% and PVA with MW of 67,000 was used for all the coating solutions).





**Supplementary Fig. 16** OTR of films before and after 50, 100, and 200 flex of PET, 10 wt% PVA coated PET, and PET film coated with 6 wt% LDH and 4 wt% PVA coating solution (PVA with MW of 67,000 was used for all the coating films). Error bar represents the standard deviations of more than 2 measurements. Source data are provided as a Source Data file.


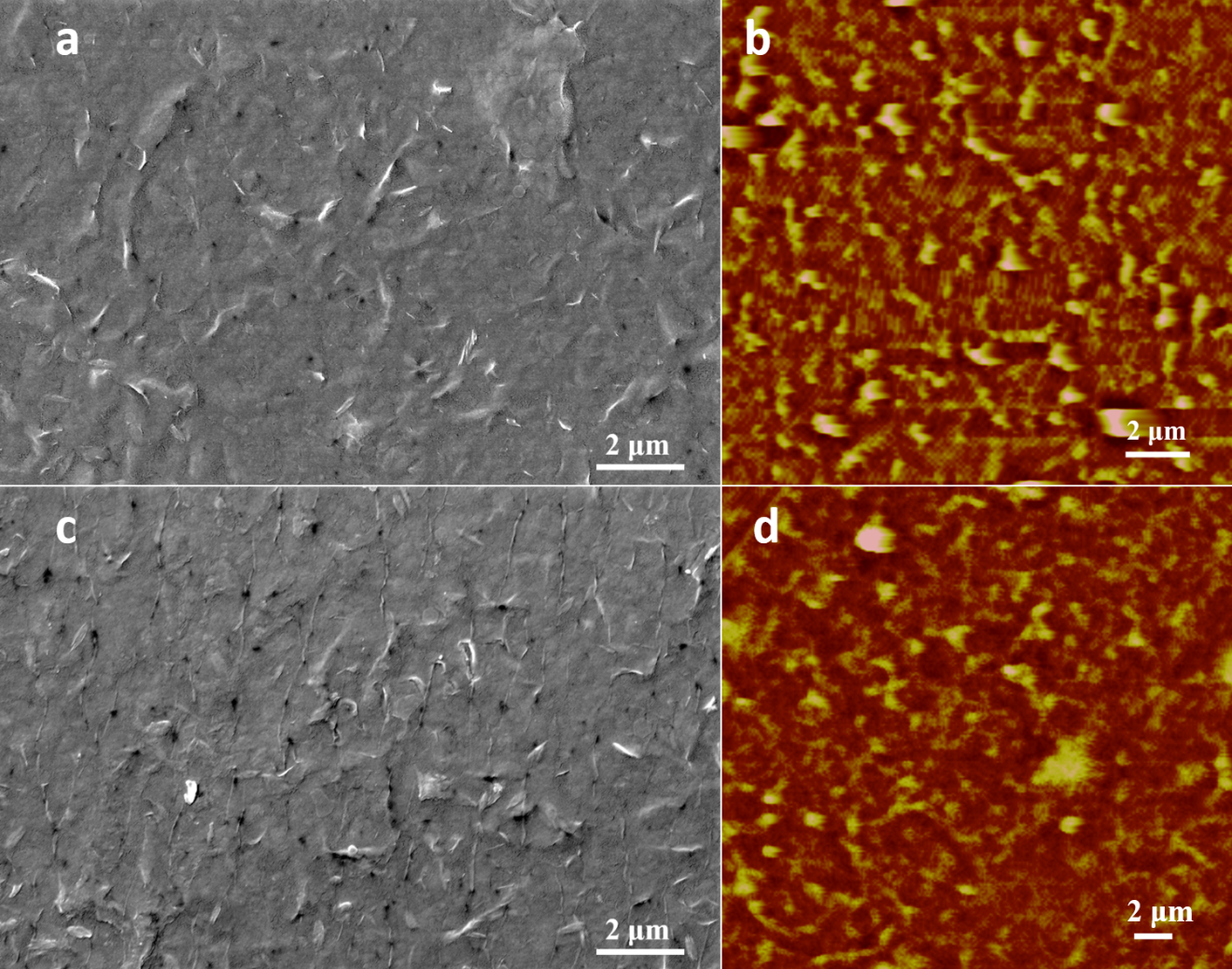


**Supplementary Fig. 17** SEM and AFM images of coating film surface (sample discussed in fig. S16) before (**a** and **b**) and after 200 flex (**c** and **d**) showing smooth surface.


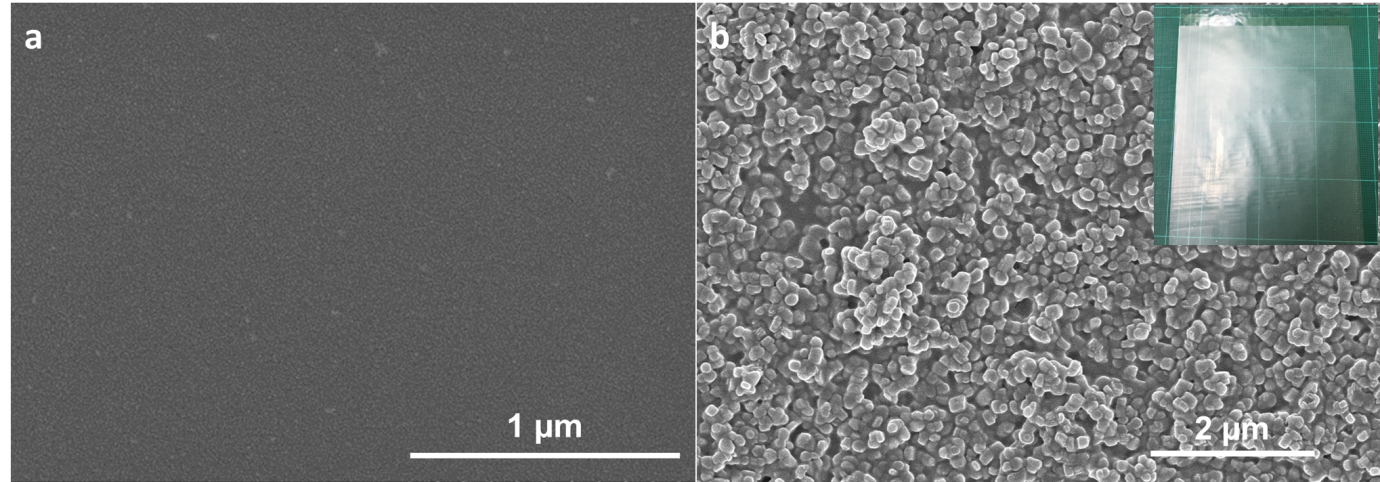


**Supplementary Fig. 18** SEM images of film surface coated with PVA (**a**) and original LDH/PVA (**b**) showing rough surface compared to the reconstructed LDH coated films (b inset shows that the film is opaque). PVA with MW of 67,000 was used for all the coating films.

**Supplementary** **Table 6** Barrier properties of the coated films. STP, standard temperature and pressure (PVA with MW of 67,000 was used for all the coating films).

| Samples^a^ | LDH (wt%) | PVA (wt%) | Total solids  (wt%) | Coating thickness  (nm) | OTR  [cc·m^-2^·day^-1^] | O_2_ permeability of coated barrier film  [10^-16^cm^3^(STP)·cm·cm^-2^·s^-1^·Pa^-1^] | BIF^b^ |
| --- | --- | --- | --- | --- | --- | --- | --- |
| PET(12) | - | - | - | - | 133.50 | 18.3000 | - |
| PVA-5 wt%-24^c^ | 0 | 5 | 5 | 890 ± 32 | 18.25 | 2.6000 | 7 |
| 5 wt%-60% LDHs-24^c^ | 3 | 2 | 5 | 665 ± 33 | 0.044 | 0.0063 | 2908 |
| 5wt%-80% LDH-24^c^ | 4 | 1 | 5 | 891 ± 42 | 0.042 | 0.0062 | 2959 |
| 7wt%-60% LDH-24^c^ | 4.2 | 2.8 | 8 | 1000 ± 47 | <0.005 | 0.0007 | 24640 |
| 10 wt%-60% LDH-24^c^ | 6 | 4 | 10 | 1103 ± 21 | <0.005 | 0.0007 | 24452 |
| 5wt%-60% LDH-6^d^ | 3 | 2 | 5 | 92 ± 10 | 1.92 | 0.2652 | 69 |
| 5wt%-60% LDH-12^e^ | 3 | 2 | 5 | 295 ± 14 | 0.21 | 0.0298 | 615 |
| 5wt%-60% LDH-12-T^f^ | 3 | 2 | 5 | 690 ± 20 | 0.041 | 0.0059 | 3079 |
| 5wt%-60% LDH-40^g^ | 3 | 2 | 5 | 1845 ± 33 | 0.036 | 0.0057 | 3213 |
| Literature | | | | | | | |
| PET(180)-LDHs^2^ | - | | | 149 | <0.005 | 0.0103 | 1685 |
| PET(180)-LDHs^3^ | - | | | 360 | <0.005 | 0.0103 | 1683 |
| PET(179)-MMT^4^ | - | | | 82.6 | <0.005 | 0.0102 | 1719 |
| PET(125)-GO^5^ | - | | | 1×10^4^ | <0.005 | 0.0077 | 2120 |
| Commercial metallized PET(12)^6^ | - | | | 42 | 0.25 | 0.0349 | 678 |

**^a^**The value inside the parentheses is the thickness of substrate PET films in μm; **^b^**Barrier improvement factor (BIF) (which is defined as Ps/Pt, where Ps is the permeability of the substrate and Pt is the permeability of the coated substrate); ^c^24 denotes the coating gap is 24 μm. ^c,d,e,g^24, 6,12, and 40 denotes the coating gap in μm. ^f^The sample is coated twice with 12 μm coating gap rod.

**Supplementary References**

1 Wyman, J. The Dielectric Constant of Solutions of Dipolar Ions. *Chem. Rev.* **19**, 213-239 (1936).

2 Dou, Y. *et al.* Transparent, Ultrahigh‐Gas‐Barrier Films with a Brick–Mortar–Sand Structure. *Angew. Chem. Int. Ed.* **54**, 9673-9678 (2015).

3 Pan, T. *et al.* Remarkable oxygen barrier films based on a layered double hydroxide/chitosan hierarchical structure. *J. Mater. Chem. A* **3**, 12350-12356 (2015).

4 Priolo, M. A., Gamboa, D., Holder, K. M. & Grunlan, J. C. Super gas barrier of transparent polymer− clay multilayer ultrathin films. *Nano Lett.* **10**, 4970-4974 (2010).

5 Chen, J.-T. *et al.* Enhancing polymer/graphene oxide gas barrier film properties by introducing new crystals. *Carbon* **75**, 443-451 (2014).

6 Jamieson, E. & Windle, A. Structure and oxygen-barrier properties of metallized polymer film. *J. Mater. Sci.* **18**, 64-80 (1983).
